# Preclinical Studies on Convalescent Human Immune Plasma-Derived Exosome: Omics and Antiviral Properties to SARS-CoV-2

**DOI:** 10.3389/fimmu.2022.824378

**Published:** 2022-03-24

**Authors:** Neslihan Pakize Taşlı, Zeynep Burçin Gönen, Oğuz Kaan Kırbaş, Nur Seda Gökdemir, Batuhan Turhan Bozkurt, Buse Bayrakcı, Derya Sağraç, Ezgi Taşkan, Sevda Demir, Nur Ekimci Gürcan, Melike Bayındır Bilgiç, Ömer Faruk Bayrak, Hazel Yetişkin, Büşra Kaplan, Shaikh Terkıs Islam Pavel, Gökçen Dinç, Müge Serhatlı, Gamze Çakırca, Ahmet Eken, Vedat Aslan, Mehmet Yay, Musa Karakukcu, Ekrem Unal, Fethi Gül, Kemal Erdem Basaran, Yusuf Ozkul, Fikrettin Şahin, Olcay Y. Jones, Şaban Tekin, Aykut Özdarendeli, Mustafa Cetin

**Affiliations:** ^1^ Faculty of Engineering, Yeditepe University, Istanbul, Turkey; ^2^ Oral and Maxillofacial Surgery, Genome and Stem Cell Centre, Erciyes University, Kayseri, Turkey; ^3^ Faculty of Medicine, Yeditepe University, Istanbul, Turkey; ^4^ Faculty of Medicine, Erciyes University, Kayseri, Turkey; ^5^ Vaccine Research and Development Application and Research Center, Erciyes University, Kayseri, Turkey; ^6^ The Scientific and Technological Research Council of Turkey (TÜBITAK) Marmara Research Centre Energy Institute, Kocaeli, Turkey; ^7^ Department of Molecular Biology and Genetics, Faculty of Science, Gebze Technical University, Kocaeli, Turkey; ^8^ Department of Biology, Faculty of Science, Erciyes University, Kayseri, Turkey; ^9^ Gevher Nesibe Genome and Stem Cell Institute, Erciyes University, Kayseri, Turkey; ^10^ Antalya Training and Research Hospital, Antalya, Turkey; ^11^ Department of Anesthesiology and Reanimation, School of Medicine, Marmara University, Istanbul, Turkey; ^12^ Division of Rheumatology, Department of Medicine, George Washington University School of Medicine and Health Sciences, Washington, DC, United States; ^13^ Medical Biology, Department of Basic Medical Sciences, University of Health Sciences, Istanbul, Turkey

**Keywords:** exosome, extracellular vehicles (EVs), COVID-19, SARS-CoV-2, convalescence plasma, viral treatment

## Abstract

The scale of the COVID-19 pandemic forced urgent measures for the development of new therapeutics. One of these strategies is the use of convalescent plasma (CP) as a conventional source for passive immunity. Recently, there has been interest in CP-derived exosomes. In this report, we present a structural, biochemical, and biological characterization of our proprietary product, convalescent human immune plasma-derived exosome (ChipEXO), following the guidelines set forth by the Turkish Ministry of Health and the Turkish Red Crescent, the Good Manufacturing Practice, the International Society for Extracellular Vesicles, and the Gene Ontology Consortium. The data support the safety and efficacy of this product against SARS-CoV-2 infections in preclinical models.

**Graphical Abstract:**
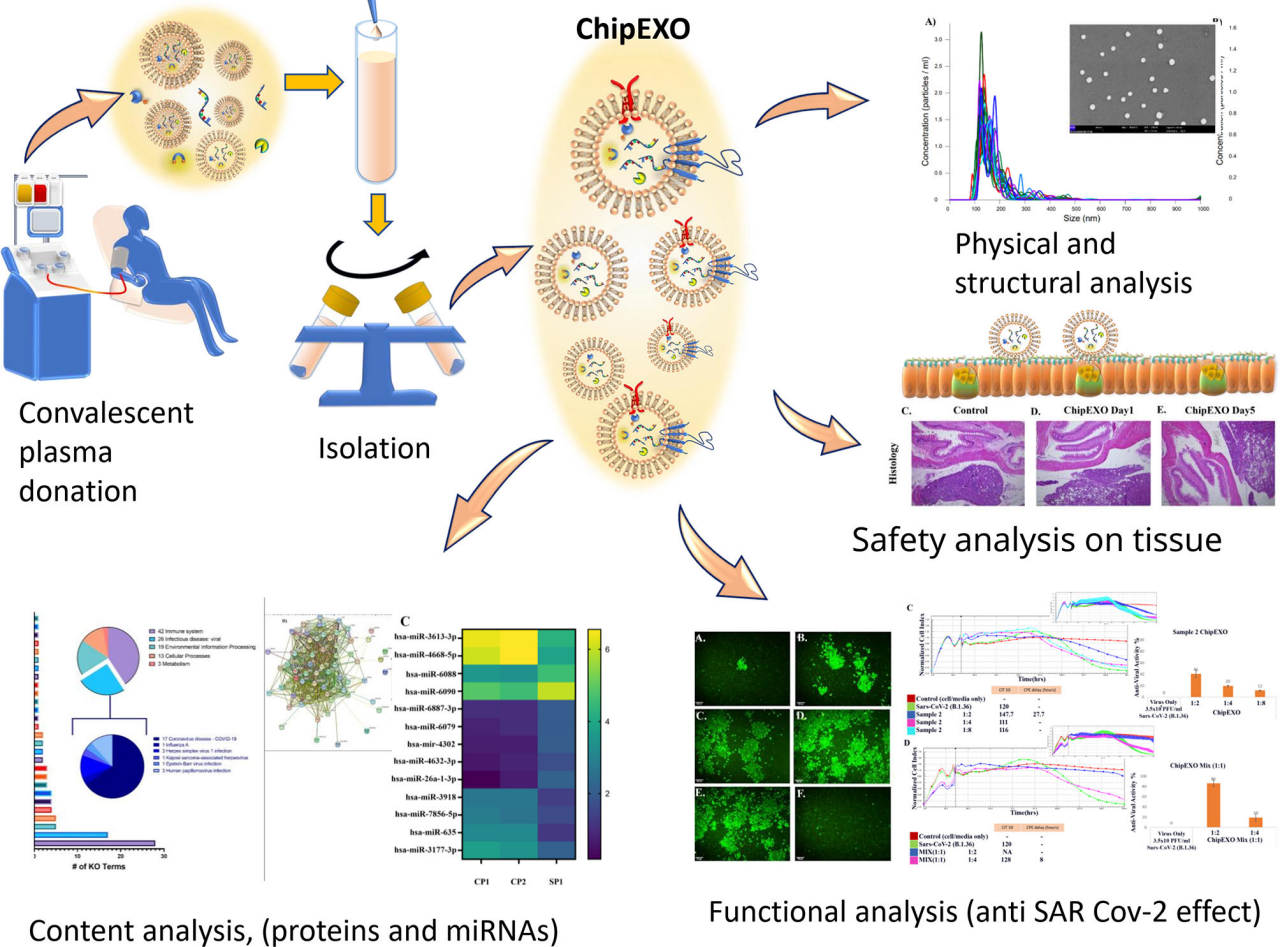


## Introduction

The coronavirus disease 2019 (COVID-19) pandemic has posed an unprecedented need for new antiviral therapeutics that are safe, effective, and readily available for large populations. Severe acute respiratory syndrome coronavirus 2 (SARS-CoV-2), the causative agent of COVID-19, is an airborne disease targeting the lung epithelial cells resulting in viral pneumonia in about 20% of the infected (1, 2). This is the major cause of mortality—so far, 4.5 million worldwide—due to the development of acute respiratory distress syndrome that involves inflammatory cascades and endothelial damage (3). As a result, any formulation of new treatment regimens to diminish the viral load and control lung inflammation has been the global focus as the mortality remains at 10% among those hospitalized (4).

Since the early days of the pandemic, many countries have been engaged in large-scale operations to collect and store convalescent serum from the survivors (5). This is considered as a historical remedy, dating back to the 19^th^ century, to provide passive immunity when needed. In fact, successful applications of convalescent plasma have been reported during the epidemics by the members of Coronoviridea, SARS, and MERS in the last two decades (6, 7). Similar observations have been published recently for the treatment of severe COVID-19 (8–12). With the advent of monoclonal antibody technology, there has been a changing landscape. This is mostly due to inherited difficulties associated with crude plasma including a wide range of donor variability for the antibody titers, fear for transmission of infectious agents, and concern for augmenting inflammatory and thrombotic cascades in a critically ill host (13). In rare events, it can also induce transfusion-related acute lung injury (TRALI), a condition likely to involve exposure to donor autoantibodies (14).

The immunotherapeutic and biologic activities of convalescent plasma, in addition to antiviral antibodies, have been discussed in recent publications (15–19). In this regard, there has been a great interest in harnessing plasma content for extracellular vesicles (EV) including exosomes for the treatment of COVID-19 (20, 21). EVs are ubiquitously produced by many cell types as membrane-bound extracellular vesicles of 30 to 150 nm in size. Through protein and RNA cargo, exosomes can convey information to distant remote cells upon uptake by endocytosis. Elegant studies by Mao et al. (22) showed that the size distribution of exosomes from patients with SARS-CoV-2 infection was similar (55 to 145 nm) but the protein content varied with infection severity. There have been experimental models to study the immunomodulatory (23, 24), tropic (25), and antifibrotic (26) activities of plasma-derived exosomes. To our knowledge, the antiviral potency of plasma-derived exosomes from COVID-19 survivors has not been reported. We now present our findings to test this concept using preclinical models.

## Methods

### Regulatory Approvals

This study was approved by the Central Scientific Review Board of the Turkish Ministry of Health and was conducted in full compliance with the rules and regulations of contributing academic institutions.

### The Viral Stocks

The hCoV-19/Turkey/ERAGEM-001/2020 strain was used in this study described in detail previously (27). B.1.36 strain was provided by the Ministry of Health, Directorate of Public Health. All viral studies were conducted at biosafety level 3 (BSL-3) laboratories at Erciyes University Vaccine Research, Development and Application Center (ERAGEM) and Genetic Engineering and Biotechnology Institutes of TUBITAK Marmara Research Center in Gebze, Turkey.

### Cell Line

Vero E6 cells (CRL-1586™, ATCC, Manassas, VA, USA) were maintained in Dulbecco’s modified Eagle’s medium (DMEM)–low glucose (Sigma, Germany) supplemented with 10% heat-inactivated fetal bovine serum (FBS) (Gibco, Waltham, MA, USA), 100 mM L-glutamine, 100 U/ml penicillin, and 100 μg/ml streptomycin (Biological Industries, USA), i.e., a complete medium. All assays were conducted on rapidly growing cells in 96-well microtiter plates, seeded as 2.5 × 10^4^ cells/100 μl media with 2% FBS/well.

Animal studies were conducted at Erciyes University after proper approval. Sixteen-week-old male Sprague–Dawley rats were maintained under routine conditions (room temperature, 12-h light cycle, fed *ad libitum*) and tested in compliance with the institutional Animal Experiment Guidelines at Erciyes University, Genome and Stem Cell Center (GENKOK).

### Convalescent Plasma Collection

Donor selection followed the rules and regulations put forward by the Turkish Ministry of Health and Turkish Red Crescent (*COVID-19 İMMÜN (KONVALESAN) PLAZMA TEDARİK VE KLİNİK KULLANIM REHBERİ*) and WHO Blood Regulators Network (WHO Blood Regulators Network (BRN) Position Paper on Use of Convalescent Plasma, Serum or Immune Globulin Concentrates as an Element in Response to an Emerging Virus*, n.d.). The donors were selected according to criteria including adult men or women (without any history of pregnancy) with PCR or serology evidence of COVID-19 in the recent past, i.e., a minimum of 2 weeks and a maximum of 16 weeks prior to collection. This is in conjunction with the donor’s current status of being negative for acute SARS-CoV-2 infection by PCR and negative for HBsAg, HCV, HIV 1-2, and syphilis by serology. The procedure for the collection of convalescent plasma in Turkey has been described (28); accordingly, 200–600 ml convalescent plasma was collected by apheresis (Trima Accel^®^) and labeled as “COVID-19 Immune Plasma” using the ISBT-128 encoding system with authorization from the Turkish Red Crescent. Witness samples were stored at −86°C as per guidelines provided by the “National Standards for Blood Service Units” and national legislation on traceability. For the current studies, two different batches of COVID-19 convalescent plasma and one batch of healthy control plasma were utilized. These samples were in storage for a minimum of 6 months after collection.

### Purification and Characterization of Plasma-Derived Exosomes

Two different methods were used for the isolation of exosomes: density cushion ultracentrifugation and aqueous two-phase system (ATPS). *Density cushion ultracentrifugation* was performed by layering 10 ml of plasma samples over 1.5 ml of 1 M sucrose solution in a 12.5-ml ultracentrifugation tube. Samples were then centrifuged at 100,000×*g* for 80 min using an SW 40i ultracentrifugation rotor (Beckman Coulter, Pasadena, CA, USA). After the centrifugation, the top layer was removed, and 1 ml of the sucrose layer was collected from the bottom carefully to ensure the exosome-containing phase remained unmixed with the contaminants of the upper phase. *ATPS isolation* of convalescent human immune plasma-derived exosomes (ChipEXOs) was performed as previously described (23, 29). Briefly, samples were mixed at a 1:1 (v/v) ratio with the isolation solution, which consists of PEG and dextran. Simultaneously, washing solution was prepared by diluting the isolation solution 1:1 (v/v) with distilled water. Samples and the washing solutions were centrifuged at 1,000×*g* for 10 min for phase separation. The upper 80% volume of the samples were discarded and then replaced with the upper 80% volumes of the washing solution and mixed *via* inversion. This process was performed twice, at the end of which the bottom phases of the samples containing the isolated exosomes were collected. Density cushion isolation provides exosome isolates with higher purity, at the expense of quantity, making it preferable to the ATPS isolation method for proteomic and transcriptomic analyses. All studies were conducted by following Good Manufacturing Practice (GMP) guidelines and under sterile conditions.

### Structural Characterization of Exosomes

#### Measurements of Physical Properties

Size distribution of exosomes was measured by nanoparticle tracking analysis (NTA) using Nanosight NS300 (Malvern Instruments, Malvern, UK). Samples were diluted in phosphate-buffered solution (PBS) to contain 25–200 particles in a frame and examined by 15 captures of 20 s each. Threshold levels were selected for each sample according to the manufacturer’s instructions.

#### Scanning Electron Microscopy

Thirty microliters of air-dried exosome suspension on a glass slide was imaged by scanning electron microscopy (Zeiss GEMINI 500, Zeiss, Oberkochen, Germany) at the Erciyes University TAUM Research Center.

#### Flow Cytometry

Exosomes were studied for surface markers by flow cytometry after coupling with aldehyde/sulfate latex beads (A37304, Invitrogen, Thermo Fisher Scientific, Waltham, MA, USA). First, 100 µl of exosome solution was mixed with 1.5 µl of bead solution and incubated for 30 min at room temperature. Then, 400 µl of PBS was added and the mixture was centrifuged at 2,700×*g* for 3 min. The pelleted bead–exosome complex was dispersed in 100 µl of 100 mM glycine solution to close the open aldehyde ends of the bead and incubated for 30 min, followed by PBS washing. Fluorescently labeled monoclonal antibodies to CD81 (349506, Biolegend, San Diego, CA, USA), TSG101 (ab209927, Abcam, Cambridge, UK), and CANX (ab203439, Abcam, Cambridge, UK) at 1:100 dilution in PBS with 1% BSA (bovine serum albumin) were added and samples were incubated overnight at 4°C. The samples were then washed twice with PBS, dispersed in 400 µl, and analyzed with the FACSCalibur flow cytometry instrument.

### Biochemical Characterization of Exosome Cargo

#### miRNA Chip Assay

MicroRNA (miRNA) expression profile was performed by Affymetrix miRNA 4.0 GeneChip assay (Affymetrix, Santa Clara, CA, USA) using GeneChip 4.0 miRNA array that contains 2,025 pre-miRNAs and 2,578 mature miRNA probes for humans. RNA samples were isolated by the TRIzol method according to the manufacturer’s RNA isolation protocol (Thermo Fisher Scientific, Waltham, MA, USA). Total RNA samples were labeled using Affymetrix FlashTag Biotin HSR RNA Labeling Kit. Briefly, 130 ng of total RNA samples were poly(A)-tailed using poly A polymerase enzyme and ATP at 37°C for 15 min, then biotinylated by ligating biotin-labeled fragment to the 3′ end using the FlashTag Biotin HSR RNA Labeling Kit following the manufacturer’s protocol. Labeled samples were hybridized on miRNA 4.0 arrays at 48°C and 60 rpm for 18 h *via* GeneChip^®^ Hybridization Oven 645 (Affymetrix, Santa Clara, CA, USA). GeneChip^®^ Fluidics Station 450 (Affymetrix, Santa Clara, CA, USA) and GeneChip^®^ Scanner 3000 7G System (Affymetrix, Santa Clara, CA, USA) were used to wash, stain, and scan the arrays, respectively. Differentially expressed microRNAs among the study groups were analyzed *via* Affymetrix^®^ Transcriptome Analysis Console software (TAC, version 4.0).

#### Proteomics

Proteomic profiling of ChipEXOs was performed by mass spectroscopy. Briefly, proteins were separated *via* 12% SDS-PAGE followed by cleanup and concentration using ReadyPrep 2-DE Cleanup Kit (Bio-Rad) according to the manufacturer’s instructions. SDS-PAGE gels were fixed in 40% methanol, 10% acidic acid, and colloidal Coomassie Brilliant Blue G-250 in distilled water (v/v) overnight. Bands of proteins were excised for in-gel tryptic digestion (Thermo Fisher). Digested peptides were preconcentrated and desalted in with a trap column and separated using an Acclaim PepMap RSLC C18 high-performance liquid chromatography (HPLC) analytical column (75 μm × 15 cm × 2 μm, 100 Å diameter, Thermo Fisher Scientific). Peptide identification was done with nLC-MS/MS using an Ultimate 3000 RSLC nanosystem (Dionex, Thermo Fisher Scientific, Waltham, MA, USA) coupled with a Q Exactive mass spectrophotometer (Thermo Fisher Scientific, Waltham, MA, USA). Full spectra mass spectroscopy of the peptides was conducted with the following settings: resolution of 70,000, scan range of 40–2,000 *m*/*z*, spray voltage of 2.3 kV, target automatic gain control of “AGC” 3 × 10^6^, and a maximum injection time of 60 ms. The identified peptides were matched to proteins using Proteome Discoverer 2.2 (Thermo Fisher Scientific, Waltham, MA, USA) with the following settings: mass tolerance of 10 ppm, MS/MS mass tolerance of 0.2 Da, mass accuracy of 2 ppm, tolerant miscarriage of 1, minimum peptide length of 6, cysteine carbamidomethylation as fixed modification, methionine oxidation as variable modification, and asparagine deamination. The final results were queried in the UniProt/Swiss-Prot database for protein identification.

### Bioinformatics

miRNA was analyzed in the Transcriptome Analysis Console (TAC) Software v4.0 program, selecting values with ±2-fold change and significance at *p <*0.05. In addition, miRNAs that were considered significant by the TAC Software v4.0 program were ontologically analyzed in the DIANA-miRPath v3.0 software (30). Venn diagram was created using the InteractiVenn software (31).

Data of all four donors were pooled together for the analyses. Functional annotation of the ChipEXO’s proteomes was made with UniProt accession numbers. Gene ontology (GO) enrichment analyses of ChipEXO’s proteomes were made using the Kyoto Encyclopedia of Genes and Genomes (KEGG) (32) and Protein Analysis Through Evolutionary Relationships (PANTHER) (33). The percentage of proteins falling under a particular term over the total number of proteins was reported for GO and KEGG ontology analyses.

### Preclinical Assessment of ChipEXO for Safety

#### Testing ChipEXO for Toxicity *In Vitro*

Two-fold dilutions of ChipEXO were added onto Vero E6 cells seeded in 96-well E-plate of the xCELLigence RTCA MP device (Agilent Technologies, Santa Clara, CA, USA) in triplicates. Throughout the experiment, the instrument was placed in a cell culture incubator at 37°C with 5% CO_2_ and was operated through a cable-connected external control unit. The assay is based on electrical impedance measured every 15 min; the electrical conductivity is converted to the unitless cell index (CI) parameter by xCELLigence RTCA Software Pro; a higher CI value indicates increased cell viability/health, whereas a lower value indicates cell death/unhealthy.

#### Testing ChipEXO for Toxicity *In Vivo*

Exposure of rats to ChipEXO was investigated as follows: unsedated healthy rats (*n* = 4 treated and *n* = 2 control)—held in upright vertical position and neck in hyperflexion—were exposed to ChipEXO (100 µl of stock solution) through intratracheal instillation over 2–3 s. Controls receive saline only. On the day of treatment, rats (*n* = 1 control; *n* = 2 study) were tested for barometric whole-body plethysmography (WBP, Buxco Systems, USA) modified for continuous flow. A constant gas flow input (6 L/min) is delivered with a mass flow controller (MFC-4, Sable Systems, North Las Vegas, NV, USA) to a gas mixer connected upstream of the chambers and gas flow output through a hole attached to the WBP cage. This allows to isolate and measure the changes in the chamber pressure from breathing by input and output impedances relative to the atmospheric pressure. For the measurement of ventilation (V), respiratory frequency (fR), and tidal volume (Vt), the rat was weighed and sealed into the WBP chamber. After the first 30 min to allow acclimation to 21% O_2_, with a constant 0.03% CO_2_ balanced N_2_, the rat was exposed to a constant flow of 21% O_2_ for 60 min. During this period, raw data were collected every 15 min, analyzed for fR, Vt, and V, and normalized to body mass [ml/(min*kg)] as described in Drorbaugh and Fenn (34) and Jacky et al. (35). Rats were then sacrificed on day 1 and day 5 post-treatment for histopathology examination of the lung and airway.

### Functional Studies to Assess the Antiviral Properties of ChipEXO

#### Titration of SARS-CoV-2

The functional studies were based on the hCoV-19/Turkey/ERAGEM-001/2020 strain as previously described (27). The viral titer was determined as tissue culture infective dose 50% (TCID50) and focus forming assay (FFA) per published methods (27, 36). *TCID50*: Vero E6 cells were seeded in 96-well plates in complete medium and incubated for 18–24 h at 37°C. Upon confluency, 10-fold serial dilutions of the virus were added to the wells in triplicates. After incubation for 1 h at 37°C with shaking, the virus inoculum was removed and the cells were washed with PBS. The plates were incubated for 5 days in 5% CO_2_, at 37°C. The cytopathic effects (CPE) were determined by inverted microscopy and TCID50 was calculated according to the Reed and Muench method (Reed et al., n.d.). *FFA*: Cell monolayers were exposed to the virus as described above for 1 h at 37°C, followed by removal by PBS washing. This was followed by the addition of a fresh medium containing 1% CMC (carboxymethyl cellulose) and incubation at 37°C with 5% CO_2_ for 24 h. Cells were then fixed with 10% neutral buffered formaldehyde at room temperature for 20 min, permeabilized with 0.1% Triton X-100 in PBS for 20 min while gently rocking, and blocked with 5% skim milk in PBS. Human antibody to SARS-CoV-2 nucleocapsid protein (1:2,500) (GenScript; HC2003) in TBST (100 mM Tris–HCl pH 8.0, 1.5 M NaCl, 1% Tween 20) was added for an hour at 37°C followed by three washes with TBST. Goat anti-human IgG conjugated to fluorescein isothiocyanate (FITCH) (1:1,000) (SouthernBiotech, USA) was added, and cells were incubated for another hour followed by three washes with TBST and once with distilled water. The antibody-labeled cells were detected and analyzed by immunofluorescence microscopy (Leica Microsystems, Wetzlar, Germany). The fluorescent foci in each well were counted, and the virus titers were calculated and expressed as fluorescent focus units (FFU) per ml as described previously (37). The results of TCID50 and FFA guided the viral dose used in the functional testing of ChipEXO as described below.

#### Assessment of the Antiviral Properties of ChipEXO by CPE

Two-fold diluted exosomes were mixed with the hCoV-19/Turkey/ERAGEM-001/2020 strain of SARS-CoV-2 at a fixed dose of 100 TCID50 and incubated at 37°C for 1 h. The mixtures were then added onto the cells in triplicates. After absorption for 1 h at 37°C, the cells were washed with PBS and further incubated (in media with 2% FBS) for 5 days in 5% CO_2_ at 37°C for CPE under an inverted microscope.

#### Assessment of the Antiviral Properties of ChipEXO by FFA

Mixtures of ChipEXO at 2-fold serial dilutions and hCoV-19/Turkey/ERAGEM-001/2020 strain of SARS-CoV-2 at a fixed dose of 100 FFU were incubated at 37°C for 1 h. The mixtures were then added in triplicate to confluent Vero E6 cell monolayers. After absorption for 1 h at 37°C, the supernatants were removed and the cells were washed with PBS. The cell monolayers were overlaid with a medium containing 1% CMC and then incubated at 37°C with 5% CO_2_ for 24 h. The remaining steps of FFA were performed as described above for a final readout under immunofluorescence microscopy (Leica, UK). The controls included mock-infected and/or mock-treated wells.

#### Assessment of the Antiviral Properties of ChipEXO by Real-Time Tracking of Viral CPE

Progression of the B.1.36 strain of SARS-CoV-2 in Vero E6 cells in the presence or absence of ChipEXO was followed by real-time measurement of CPE using xCELLigence RTCA MP system (Agilent Technologies, Santa Clara, CA, USA) as described above. Two-fold dilutions of ChipEXO from different donors were prepared and tested individually or tested as a 1:1 mixture (by volume) of the two. First, the cells were incubated for 24 h in the xCELLigence RTCA MP device then exposed to 3.5 × 10^5^ PFU/ml SARS-CoV-2 virus for 1 h. Without a change of media, ChipEXO was added to the wells, and cells were incubated for 160 h at 37°C with 5% CO_2_. Controls included wells with virus, exosome, or media alone.

### Statistical Analysis

All experimental data in this study were analyzed using GraphPad Prism 8 software. One-way ANOVA was used to evaluate the statistical significance of results at a *p*-value less than 0.05, which is considered an alpha value. Each experiment was repeated three times.

## Results

### Donor Information

The study utilized plasma from five donors selected according to the Turkish Ministry of Health, Turkish Red Crescent, and WHO guidelines. None of the subjects had comorbid conditions or health concerns; none was on any type of medications or supplements. COVID-survivor donors were hospitalized for viral pneumonia and received supplemental oxygen and oral favipiravir treatment (38). The plasma collection was carried out between 21 and 30 days after complete resolution of all symptoms, respectively; at that time, two donors had negative PCR and positive anti-COVID-19 serology. None of the subjects received the COVID vaccine prior to plasma collection.

### Characterization of Convalescent Plasma Exosomes

The exosome start solution out of 200 ml plasma was prepared in a 20-ml volume with normal saline (0.9% NaCl). The concentration of nanoparticles within these stock solutions was similar among the donors with readings at 2.07–3.52 × 10^11^/ml. The stock solution was stored at 4°C and tested within 2 days. Exosomes were isolated from each plasma stock using two different isolation methods (density cushion ultracentrifugation and ATPS). Size distribution, SEM micrographs, and flow cytometry results of exosomes isolated with both methods were similar to one another. Isolated exosomes were characterized based on MISEV criteria (39).

**Figure 1:**
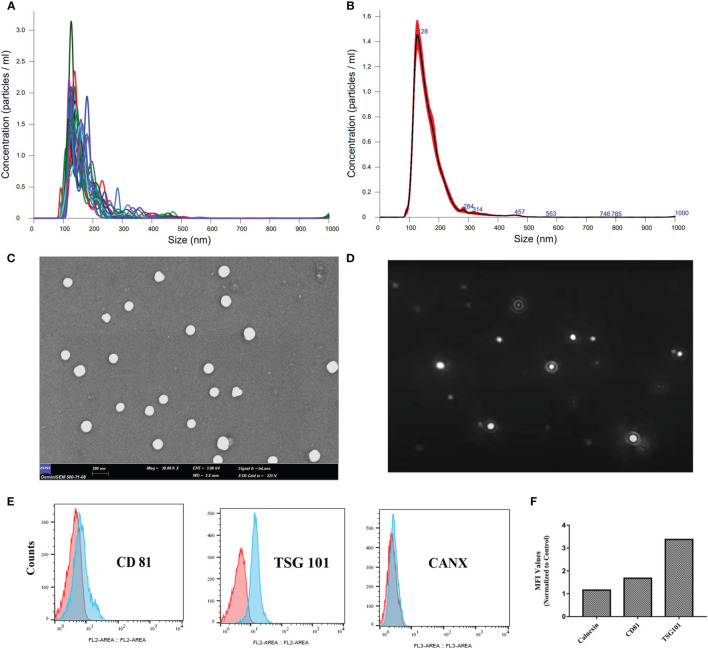
Characterization of convalescent human immune plasma-derived exosomes (ChipEXOs). **(A)** Individual size distribution measurements. **(B)** Mean size distribution measurements. **(C)** SEM micrograph. **(D)** Dynamic light scattering image. **(E)** Bead-assisted flow cytometry measurements of key exosome markers (CD81 and TSG 101) and a negative control marker (CANX). **(F)** Geometric MFI values are provided above the peaks. MFI, mean fluorescent intensity.

Physical characterization of the ChipEXOs was performed with NTA and scanning electron microscopy for size, concentration, and morphology. The mean size distribution of plasma-derived exosomes of the donors was 114 ± 15.6 nm, resulting in a 95% confidence interval (**Supplementary Table 1**). The size distribution/concentration was homogeneous with a single peak when graphed (**Figures 1A, B**).

The morphology of isolated exosomes was uniform and spherical as shown by the SEM images in **Figure 1C**. Brownian motion measurements of the EVs were used in determining size and concentration measurements (**Figure 1D**). The bead-assisted flow cytometry profile of the exosomes was positive for the known exosome markers TSG101 and CD81; staining for intracellular CANX was negative as expected (negative control) (**Figures 1E, F**).

#### Transcriptomics

**Figure 2:**
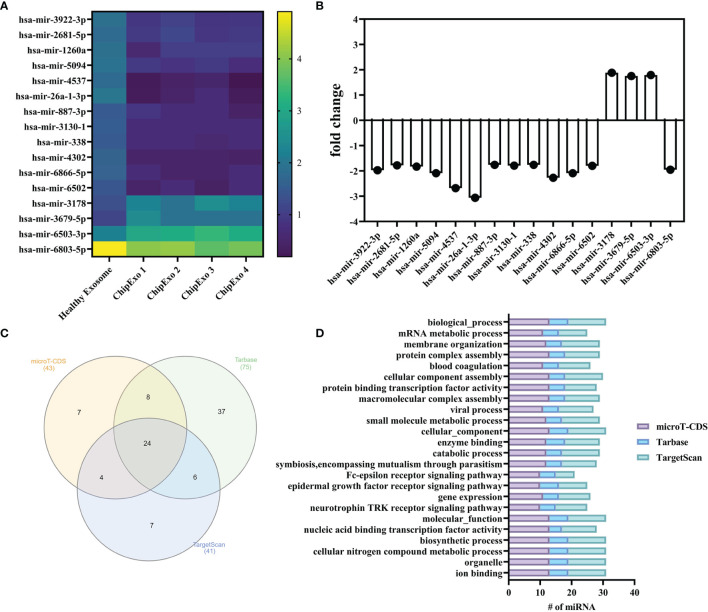
miRNA analysis of ChipEXOs. **(A)** Heatmap demonstration of miRNA signals from two different sources of ChipEXOs and healthy-EXOs. **(B)** Enrichment and depletion of different miRNAs between the ChipEXO and healthy-EXO samples. **(C)** Venn diagram of GO pathways from three different databases (microT-CDS, TarBase, and TargetScan) of miRNAs. **(D)** GO pathway graph of miRNA found in all three databases.

**Figure 2** summarizes the results of miRNA profiles found in ChipEXO prepared from four different donors in comparison to plasma exosomes from a healthy control. Accordingly, the expression profile of ChipEXO significantly differed for 13 miRNA compared with healthy control. The data on these 16 miRNAs are shown in **Figures 2A, B**, as heatmap and bar graph, based on the signals generated by the present rates and fold change rates, respectively. Furthermore, these 16 miRNAs were associated with 16 different GO pathways that were shared by all three miRNA databases (microT-CDS, TarBase, and TargetScan) as shown in the Venn graph (**Figure 2C**); these pathways are listed in **Figure 2D**.

#### Proteomics

**Figure 3:**
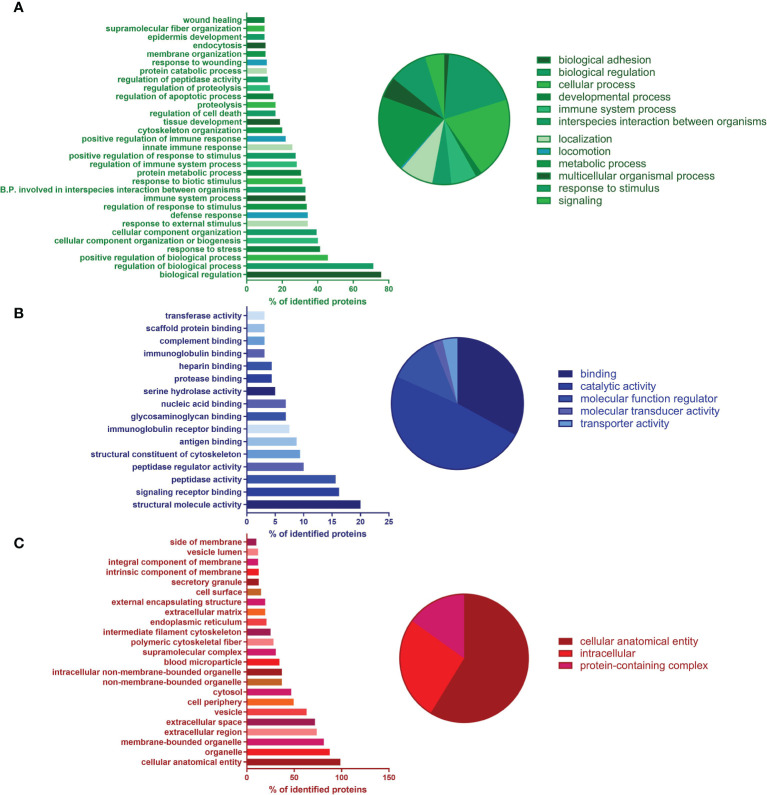
Proteomics of ChipEXO; Gene Ontology (GO) analysis according to functional enrichment networks: **(A)** biological process (green), **(B)** molecular function (blue), and **(C)** cellular component (green).

GO enrichment was used to analyze the proteomic composition of ChipEXOs (33, 40). As shown in **Figure 3**, GO annotations showed enrichment of proteins under three main domains: those associated with the biological process, molecular function, and cellular component. The proteins under the biological process included those associated with immune activation and modulation; terms such as “response to symbiont” (a.k.a. response to the virus), “cytolysis by a host of symbiont cells,” and “killing by a host of symbiont cells” included C4b-binding protein (C4BP) alpha and beta chains, apolipoprotein L1, histidine-rich glycoprotein, and prothrombin (**Figure 3A**). The proteins under “molecular function” annotated five proteins under “complement binding” and four under “immunoglobulin binding,” for the enrichment of 80.39-fold and 58.72-fold, respectively, compared with the expected number of proteins based on the PANTHER reference list of the *Homo sapiens* gene database (**Figure 3B**). In proteins under “cellular compartment,” GO term analysis showed enrichment of proteins associated with extracellular vesicles, exosomes, and plasma membrane elements (**Figure 3C**). Those directly under the term “extracellular exosome” made up 26.1% of the identified proteins. The samples did not contain any contaminants that can be associated with exosome preparations, i.e., nuclear or mitochondrial proteins. The complete proteome is provided in the **Supplementary Material**.

**Figure 4:**
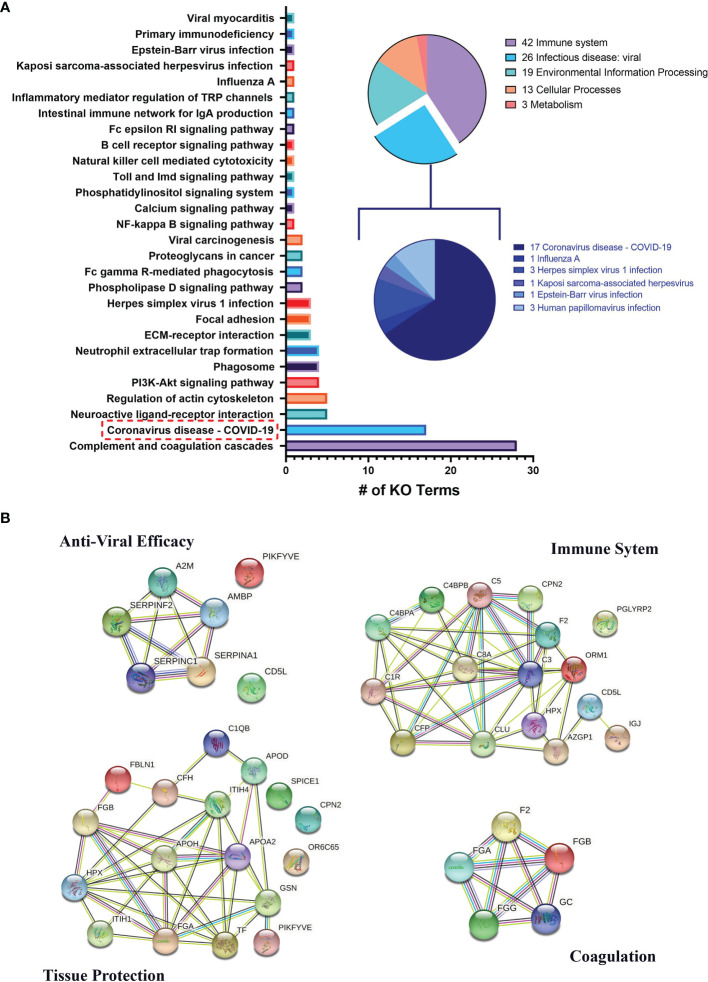
**(A)** KEGG Ontology (KO) data of proteomic analysis of ChipEXO. **(B)** STRING relation scheme of the ChipEXO protein-enriched pathway.

In addition to PANTHER, we also used KEGG to analyze the proteome (32). Notably, 28 KEGG Ontology (KO) terms (13.8% of all terms) were associated with “Complement and coagulation cascades.” Furthermore, 17 (8.4% of all terms) were associated directly with “Coronavirus disease—COVID-19” (**Figure 4A**); within these 17 KO terms, there were 64 unique proteins. Please find the full list of these 64 COVID-19-associated proteins in **Supplementary Table 5** and the STRING relation scheme of ChipEXO proteins’ functionally enriched pathways (**Figure 4B**).

### Safety and Efficacy of ChipEXO in Preclinical Models

**Figure 5:**
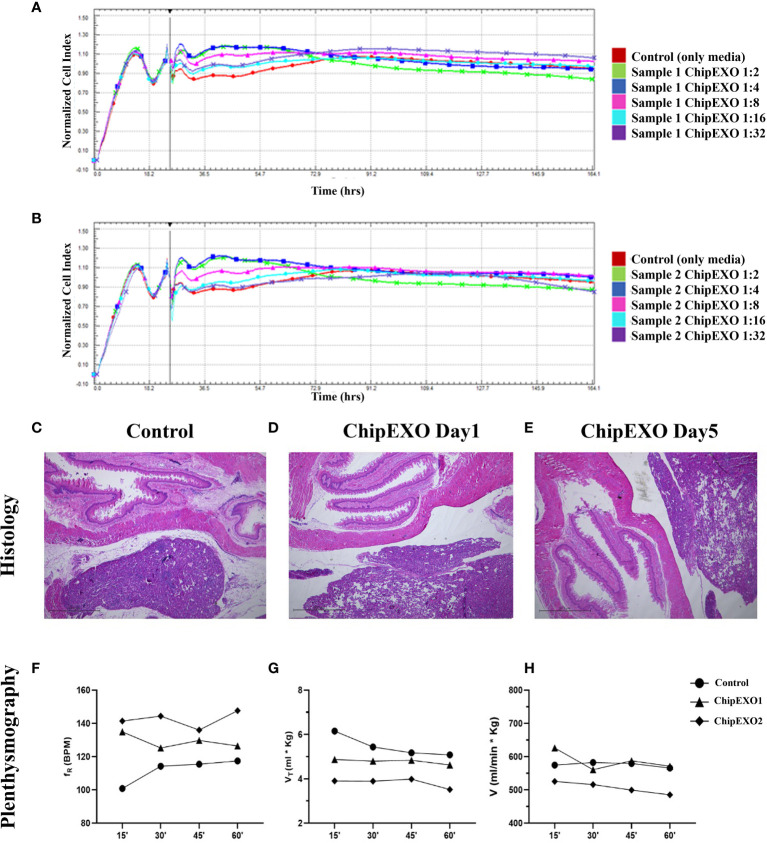
Cytotoxicity of ChipEXOs from donor samples—sample 1 **(A)** and sample 2 **(B)**—on Vero E6 cells by real-time cytotoxicity assay on RTCA MP real-time cell analysis system. The data in the figure have been adjusted to the time point when the virus was added to the experiment. Histology: **(C)** control and **(D)** day 1 and **(E)** day 5—the exosome-administered animal showed no pathological changes in lung tissue of hematoxylin–eosin (H&E)-stained sections; ×4 magnification. Plethysmography: time activity for intratracheal instillation ChipEXOs. Exposure to normoxia (21.0% O_2_) groups does not affect respiratory frequency (fR) **(F)**. Tidal volume (Vt) **(G)** and minute ventilation (V) **(H)** during whole-body plethysmography measurement. Bonferroni after repeated measures two-way ANOVA; all data presented as mean ± SEM: *N* = 1 for the control group and *N* = 2 for the exosome group. (The *x*-axis shows time in minutes). The difference for the plethysmography data points between the treated and untreated control was not statistically significant.

**Figure 5** summarizes the safety evaluation of ChipEXO. *In vitro*, incubation of cells in the presence of ChipEXO did not cause cellular toxicity by visual exam under the inverted microscope for CPE (data not shown) or by automated xCELLigence system for cell viability (**Figures 5A, B**). *In vivo*, exposure of rats to ChipEXO did not cause immediate or delayed respiratory distress or allergic reaction. The tissue histopathology of airways and lung parenchyma did not show any signs of inflammation, necrosis, or thrombosis. Interestingly, a trend of improvement in lung functions was noted in rats treated with high-dose ChipEXO compared with mock-treated controls.

**Figure 6:**
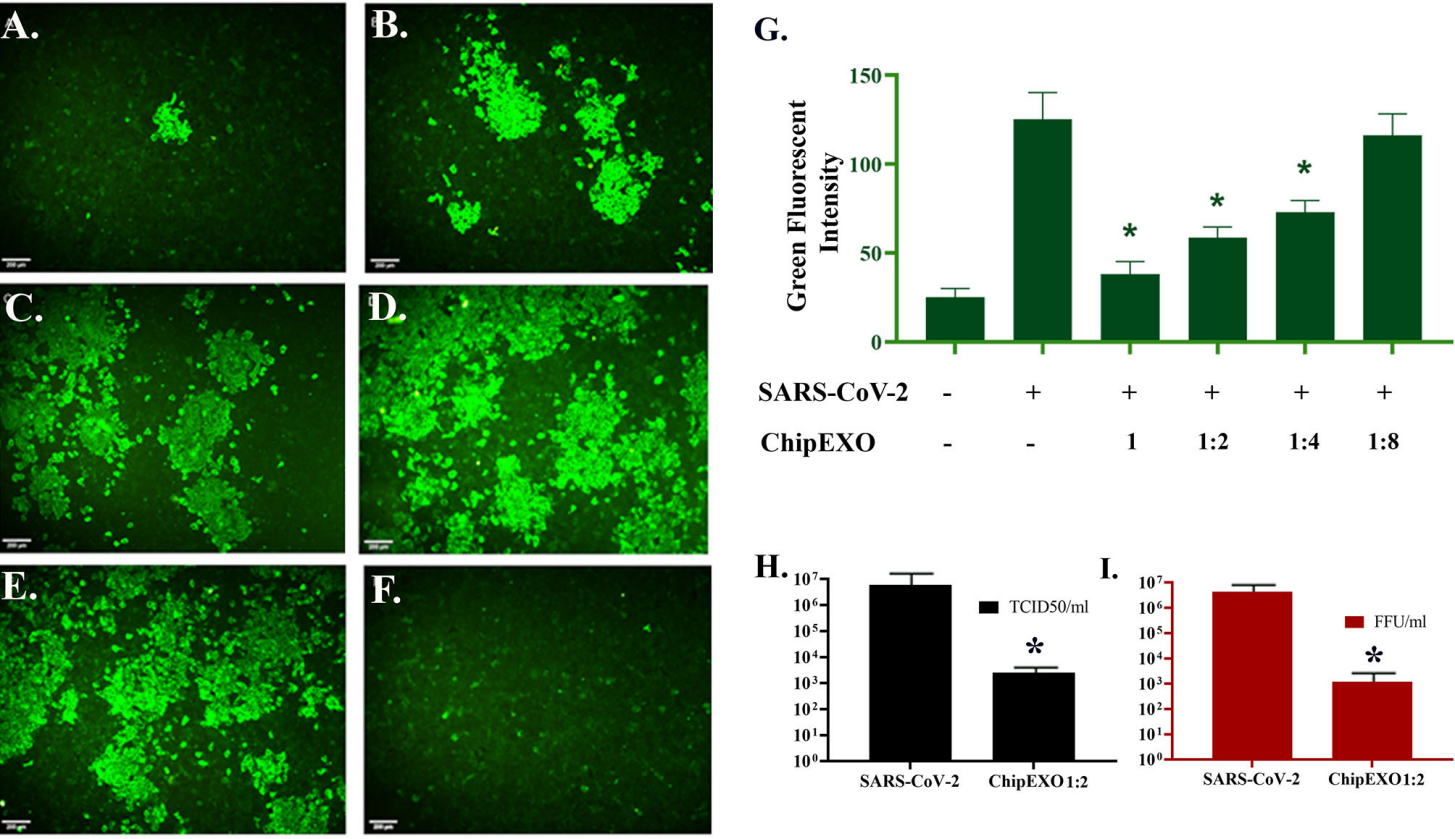
Antiviral activity of ChipEXOs. Undiluted **(A)**, 1/2 **(B)**, 1/4 **(C)**, and 1/8 **(D)** dilutions of ChipEXOs were mixed with 100 FFU of the SARS-CoV-2 and incubated at 37°C for 1 h. Infected non-treated **(E)** and mock-infected **(F)** controls were also included. **(G)** Bar graphical demonstration of green fluorescent levels of the virus antigen. **(H)** Comparison of TCID50 values of the virus-infected control and ChipEXO-treated cells. **(I)** Comparison of FFU values of virus-infected control and ChipEXO-treated cells. The antibody-labeled cells were detected and analyzed by immunofluorescence microscopy (Leica, DFC450C). Scale bars = 200 μm (*p < 0.05).

**Figure 7:**
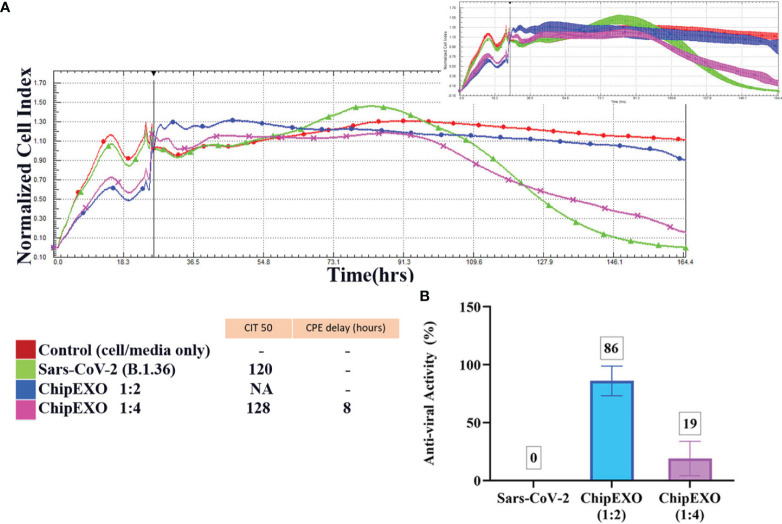
The antiviral efficacy of ChipEXO was evaluated using the xCELLigence RTCA MP real-time cell analysis equipment.The xCELLigence system’s cell index (CI) for Vero E6 cells in media (red line), or after viral inoculation (3.5 × 105 PFU/ml), or alone (green line) **(A)**. CI in the presence of virus and ChipEXO from two different concentrations, respectively (1/2 and 1/4) **(B)**. In the top right corner, a smaller second graph displays the same data with the standard deviation added. The bar graph and table depicted the antiviral activity rate of ChipEXO and the CITmed and CPE delay hours, respectively. Each curve was obtained from at least three separate duplicates of normalized cell index (NCI) values.

The antiviral activity of ChipEXO was tested *in vitro* using the Vero E6 cell line by two separate assay systems, each differing for viral strains and sequence of exposure to virus and exosomes. The common findings from these assays were as follows: ChipEXO had potent antiviral properties, and the effects were dose-dependent. Briefly, **Figure 6** summarizes the results of the first assay system based on TCID50 and FFU. Here, cells were exposed to a fixed amount of viral load premixed with varying doses of ChipEXO for 1 h followed by the removal of virus and exosomes by a wash and continuing incubation in fresh media with 2% FBS for a total of 1 to 5 days. The viral titer was significantly reduced in the presence of high-dose ChipEXO (i.e., 1:2 dilution); this corresponded to a decline in TCID50/ml from 6.01 × 10^6^ to 2.55 × 10^3^ and FFU/ml from 4.3 × 10^6^ to 1.2 × 10^3^. The antiviral effect was dose-dependent and there was no detectable viral inhibition at 1/4 and 1/8 dilutions. **Figure 7** summarizes the results of the second assay system using automated xCELLigence allowing real-time data collection. Cells were exposed to SARS-CoV-2 at a fixed dose for 1 h prior to adding varying doses of ChipEXO in the wells; thus, both virus and exosomes were present in the culture media during the remaining of the assay. The antiviral activity, based on CI values, was about 40% to 50% in the presence of high-dose exosomes. Interestingly, the effects of ChipEXOs were augmented when exosomes from two donors were mixed suggesting donor-specific cargo with additive bioactivities. Again, the effect was dose-dependent.

The antiviral abilities of the ChipEXO samples of various dilutions were monitored in real-time with a 160-h incubation. The antiviral activity of ChipEXO was calculated using CITmed and CPE delay hours. All samples were normalized to the time point at which the virus was initially added, and this point was used to create the NCI (**Supplementary Figure 2A**). Using the NCI as the initial reference value, the time-lapse observed until the readings that correspond to 50% of the maximum value (i.e., CITmed) was determined in the presence of the virus alone. This allowed computing and comparing the time-lapse to reach the CIT50 value in the presence of ChipEXO. Based on CIT50 values, both ChipEXO samples delayed CPE only at 1:2 dilution, and this was for an average of 25 ± 3.8 h. The calculated antiviral activity of ChipEXO sample 1 at CITmed was 52%, 13%, and 10% (**Supplementary Figure 2B**), and that of sample 2 was 41%, 20%, and 12% (**Supplementary Figure 2C**), at 1:2, 1:4, and 1:8 dilutions, respectively. The antiviral activity of ChipEXO, however, was significantly increased (86% at 1:2 dilution) in wells treated with a 1:1 mixture of both ChipEXO samples (**Figures 7A, B**).

## Discussion

Exosomes are ubiquitous products of many cells composed of a diverse array of proteins and RNA cargo engulfed within a lipid bilayer-enclosed vesicle. They are paracrine units of information that represent a form of a dynamic adaptive complex system for intercellular communications. This is a growing field for the diagnostic and therapeutic applications of exosomes in medicine that has intensified recently with the advent of the COVID-19 pandemic.

In the current study, we characterized ChipEXOs from COVID-19 patients. The physical characteristics of the exosomes were compatible with previous reports (23, 39). ChipEXO samples were free of SARS-CoV-2 viral elements by RT-PCR (data not shown). We tested for its safety *in vitro* and *in vivo* using three different preclinical models. Most importantly, to our knowledge, this is the first report to show the anti-SARS-CoV-2 properties of these exosomes. ChipEXO prepared from different donors consistently showed suppression of viral propagation and preservation of cell viability. The biological activities were fast, potent, and dose-dependent. These results from three different readout assays were conducted independently at two different virology research centers and were found comparable to one another.

The omics data of exosomes from convalescent plasma significantly differed from those of healthy control. The miRNA profile of ChipEXO was similar between the four donors and was significant for 13 isolates through orthological and ontological studies. These 13 miRNAs led to 16 common GO definitions. When these 16 pathways are examined in detail, they overlap with the definitions of miRNAs found in the literature (41). Overall, the common theme of the miRNA profile of ChipEXO appears to center on those promoting tropism and those involved in immune regulation, most already defined in the literature (42, 43). Interestingly, two miRNAs, mir-3613-3p and mir-635, found in ChipEXO are known to inhibit type I interferon pathway (44) possibly by mechanisms involving cytidine monophosphate kinase 1 (CMPK1) as well as JAK kinases (JAK1 and JAK3) (45). Further studies are needed to determine the role and potency of ChipEXO in the control of inflammation.

The proteomics data were compatible with the miRNA findings and similar to previously published reports (46). As summarized in **Figure 4B** and **Supplementary Figure 3**, the ChipEXO cargo showed products involved in four main pathways with functional continuum against COVID-19 infection. The first of these interacting pathways is the “Immune Modulation” pathway which included both elements of the complement cascade and regulatory proteins. The second pathway is the “Angiogenesis” pathway and included proteins to prevent coagulation and vasoconstriction upon virus infection. The third pathway is the “Tissue Protection” pathway, which included proteins involved in homeostasis, tissue protection, and regeneration. The fourth group was compiled under the “Antiviral Activity” pathway, which includes serine protease inhibitors and prevents the virus from binding to receptors such as ACE2 and PIKFYVE in the cell and blocks its entry into the cell.

To further elaborate, we found enrichment of the complement proteins properdin, C1r, C5, C1q, C1QB, C4BPB, C4BPA, and C8A in the ChipEXO compared with exosomes from healthy donor plasma. Similar observations have been reported by Mao et al. (22) and Sin Man Lam et al. (47). Activation of the complement system is necessary to induce anti-SARS-CoV-2 immunity, yet it can also contribute to endothelial cell damage and multiorgan failure (48, 49). ChipEXOs included protein cargo involved in vasodilation and anticoagulation. In particular, vWF, HRG, PROS1, GC, F2, FGA, and FGB proteins, which are responsible for the expansion of vessels and new vessel formation, modulate blood coagulation and mitigate against vasoconstriction and coagulation caused by virus infection (50). Furthermore, some of the enriched proteins in the ChipEXOs, including Alpha-2-macroglobulin and Serpin peptidase inhibitor, clade C (antithrombin) (SERPINC1), are anticoagulants with potential benefits to the host’s vascular health.

Another group of proteins enriched in the ChipEXOs are those associated with tissue and organ protection. This group included apolipoproteins (APOD, APOA2, and APOH), which are responsible for lipid metabolism; inter-alpha-trypsin inhibitor heavy chain (ITIH1, 2, and 4) proteins, which are secreted by hepatocytes and have both calcium ion binding and serine-type endopeptidase inhibitor activity; and FBL1 proteins, which bind to fibrinogen and modulate platelet adhesion, also play an important role in tissue homeostasis.

Antiviral activity is the fourth pathway representing some of the proteins enriched in the ChipEXOs. Studies on exosome uptake and half-life are important to distinguish whether ChipEXOs inhibit viral entry and/or viral replication. The candidates are being actively studied to further define the mechanisms of antiviral activities of ChipEXOs. Based on the literature review, gelsolin, an actin-binding protein that can trim and remodel the cytoskeleton, may be important (51). Gelsolin deficiency or its overexpression has been shown to inhibit the entry of HIV into the NKR-CCR5 cell line (52). Another important molecule enriched in the ChipEXOs is alpha-1-antitrypsin and alpha-1-Antichymotrypsin. Alpha-1-antitrypsin has recently been reported to block SARS-CoV-2 infection of Vero E6 cells by blocking the processing of SARS-CoV-2 S protein by furin and the transmembrane serine protease TMPRSS2 (53, 54). Previous studies have demonstrated that exosomes might have antiviral activity against some viruses as shown by Kesimer et al., with exosomes derived from human tracheobronchial ciliated epithelium which inhibited influenza A virus infection of Madin–Darby canine kidney (MDCK) cells, possibly due to the presence of sialic acids on the surface of exosomes which can then bind and inhibit the entry of the virus (55). The exosomes derived from HeLa cells transfected with receptor for SARS-CoV-2 angiotensin-converting enzyme 2 (ACE2) plasmid or those isolated from COVID-19 convalescent as well as healthy donor plasma were shown to contain ACE2 and neutralize SARS coronavirus infection in culture (56, 57). Healthy and convalescent plasma-derived exosomes, however, did not contain ACE2 in our study, suggesting ACE2-independent antiviral mechanisms.

It is suggested that infusing COVID-19 convalescent plasma (CCP) containing virus-specific antibodies might provide antibody-dependent elimination of infected cells due to the passive transfer of virus-specific antibodies. However, so far, this treatment only provided minor benefits in clinical course and outcomes (58). Recent studies revealed that two factors limit the success of CCP treatments: the development of autoantibodies against type-1 interferons, the main mediators of the immune response, or the presence of non-neutralizing antibodies, which may lead to antibody-dependent enhancement (ADE) (59). The limited therapeutic benefits attributed to CCP treatment could be due to the immunologically effective exosomes, derived from cytotoxic CD8 and effector Th1 T cells, as well as from NK cells, rather than the immune antibodies present in CCP (60). Many of these exosomes are capable of recognizing antigens with adequate sensitivity and specificity and can trigger an immune modulation into the cells and act as an epigenetic inheritor response to target pathogens through RNAs (61). In this study, we show that convalescent human immune plasma-derived exosomes, dubbed as ChipEXO, show remarkable antiviral, anticoagulant, and anti-inflammatory capabilities *in vitro* and characterized the various proteins and miRNA they carry. ChipEXO has the potential to be a promising and novel therapeutic strategy for the treatment of COVID-19-mediated lung injury and acute respiratory distress syndrome pneumonia.

In summary, the results from current data provide evidence that convalescent human plasma-derived exosomes have potent antiviral properties and may offer complimentary effects to promote tissue protection and immune modulation. Based on these encouraging findings, there is an ongoing phase I/II trial on the safety and efficacy of ChipEXOs for the treatment of COVID-19 with impending respiratory failure. Further investigations are in progress to further characterize this novel therapeutic agent offering biological activities beyond any known plasma-derived product during the fight against the pandemic.

## Data Availability Statement

The data presented in the study are deposited in the ProteomeXchange Consortium via the PRIDE^70^ partner repository with the dataset identifier PXD032262.

## Ethics Statement

The studies involving human participants were reviewed and approved by the Turkish Ministry of Health. The patients/participants provided their written informed consent to participate in this study. Written informed consent was obtained from the individual(s) for the publication of any potentially identifiable images or data included in this article.

## Author Contributions

The experimental design of this study and the construction and analysis of the experiments were done by NT, ZG, OYJ, and MC. Plasma samples were collected by FG and MY. Exosome isolation and characterization were performed by NT, ZG, NSG, OK, BTB, BB, ET, DS, SD, YO, and FŞ. The preparation of the figures was structured by VA. miRNA analysis was performed by NT, NEG, MB, and ÖB. Antiviral activity was performed by AO, HY, BK, SP, GD, MS, ŞT, and GÇ. Safety assessments were performed by ZG, MS, ŞT, and GÇ. All authors wrote parts of the manuscript. Grammar correction and final writing were done by OYJ and AE. All authors read and approved the final manuscript.

## Funding

This work was supported by the Erciyes University Scientific Research Projects Coordination Unit (grant no. 9328), Erciyes University Scientific Research Foundation (grant no. TSG-2019-9644), and TÜBİTAK-T1004 (grant no. 18AG020). This study was partly funded by Yeditepe University. Publication of this study was supported in part (OYJ) by the Eleanor Naylor Dana Charitable Trust.

## Conflict of Interest

The authors declare that the research was conducted in the absence of any commercial or financial relationships that could be construed as a potential conflict of interest.

## Publisher’s Note

All claims expressed in this article are solely those of the authors and do not necessarily represent those of their affiliated organizations, or those of the publisher, the editors and the reviewers. Any product that may be evaluated in this article, or claim that may be made by its manufacturer, is not guaranteed or endorsed by the publisher.
