# Supplementary material for: Preclinical Studies on Convalescent Human Immune Plasma-Derived Exosome: Omics and Antiviral Properties to SARS-CoV-2
Source: Front Immunol. 2022 Mar 24;13:824378. doi: 10.3389/fimmu.2022.824378 (PMC8987587; doi:10.3389/fimmu.2022.824378)
Supplement: Supplementary file 1 [file DataSheet_1.pdf]

Supplementary Data

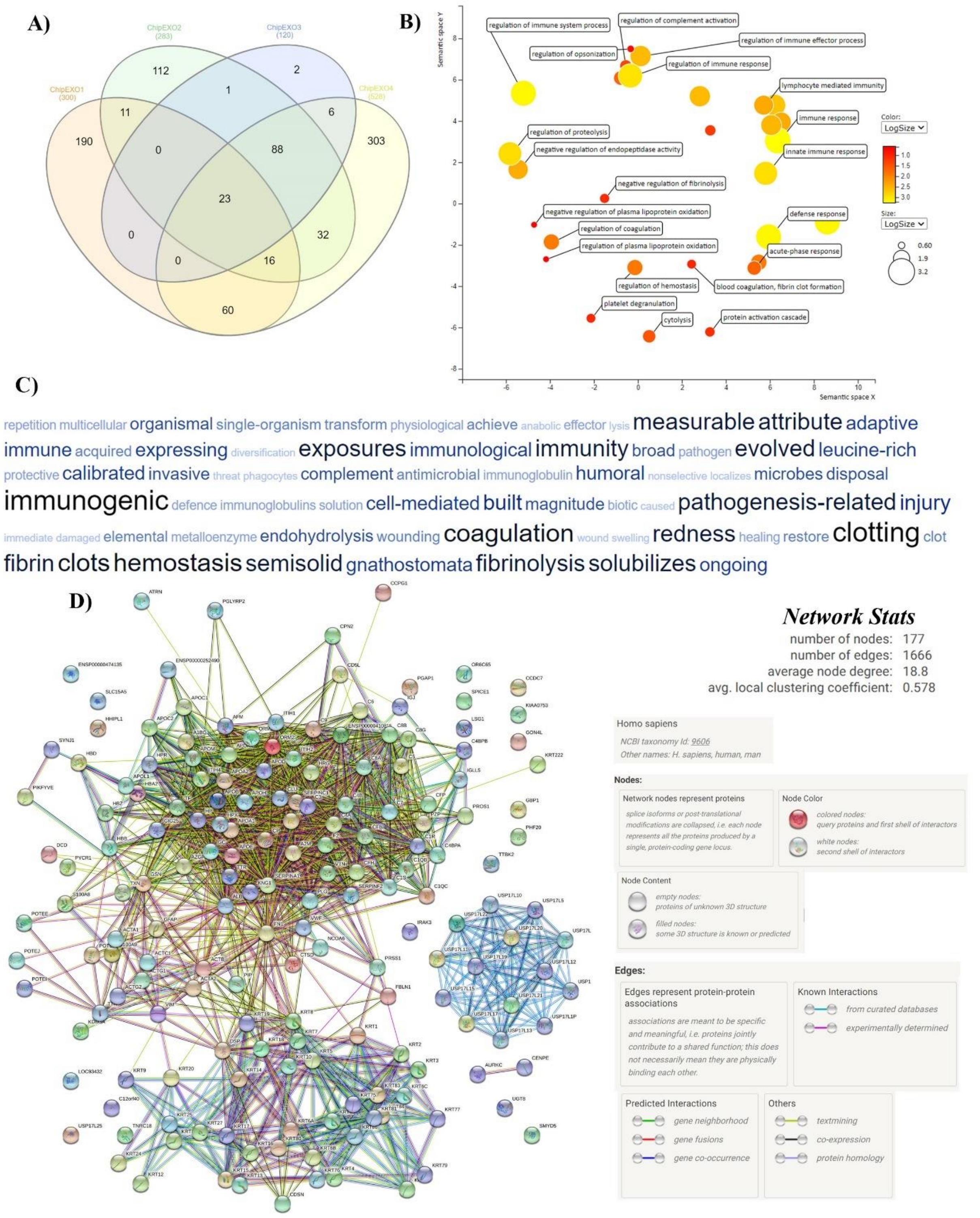

Supp Figure 1: Proteomic analysis of ChipEXO. A) Venn diagram of convalescent ChipEXO obtained from four different individuals, B) Regina graph of ChipEXO, C) Regina demonstration of ChipEXO, and D) STRING relation scheme of pooled ChipEXO proteins.

Proteomic analysis of convalescent plasma exosomes revealed total 844 unique proteins. 23 of these proteins were shared between the four samples (Supp Fig 1.A). ChipEXO proteins were enriched in GO terms associated with the immune response and clotting (Supp Fig 1.B, C). String analysis of ChipEXO proteins were also performed to show the interactions between different proteins of the proteome (Supp Fig 1.D).

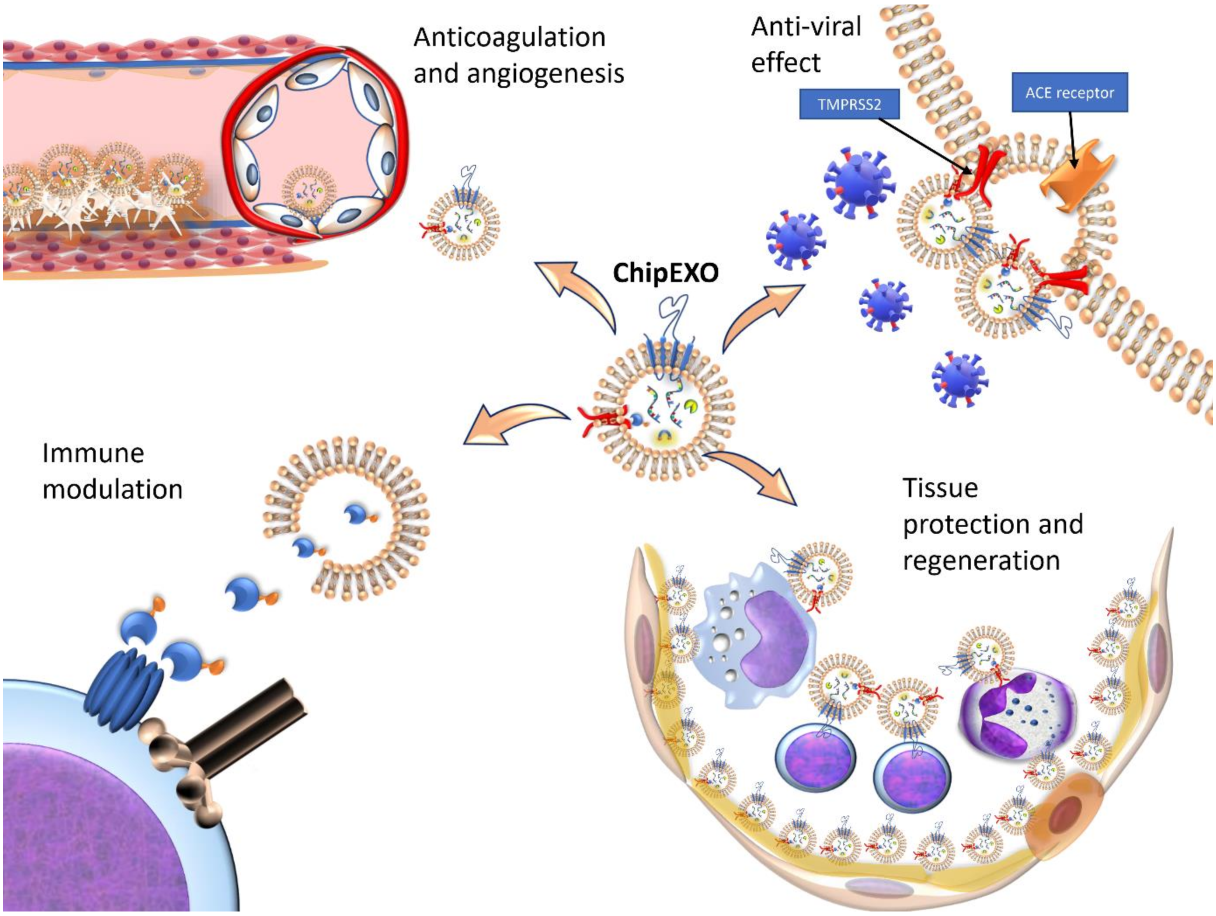

Supp Figure 2: Hypothetic mechanism of convalescent human immune plasma derived exosomes. ChipEXO cargo; showed products involved in 4 main pathways with functional continuum against Covid-19 infection.

Supplementary Table 1: Concentration and size measurements of ChipEXO with 10 technical and 4 biological replicates. SD: Standard deviation, CI: Confidence Interval, LL: Lower Limit, UL: Upper Limit

Read No	ChipEXO 1	ChipEXO 2	ChipEXO 3	ChipEXO 4
1	3,28E+11	2,7E+11	2,45E+11	2,8E+11
2	3,73E+11	2,16E+11	2,34E+11	2,7E+11
3	2,92E+11	2,47E+11	2,31E+11	2,6E+11
4	3,47E+11	2,24E+11	2,8E+11	2,8E+11
5	3E+11	2,31E+11	2,04E+11	2,5E+11
6	2,6E+11	1,68E+11	2,64E+11	2,3E+11
7	3,13E+11	2,5E+11	2,39E+11	2,7E+11
8	3,28E+11	2,02E+11	2,45E+11	2,6E+11
9	3,93E+11	2,09E+11	2,02E+11	2,7E+11
10	3,43E+11	2,34E+11	2,27E+11	2,7E+11
**Mean**	**3,28E+11**	**2,25E+11**	**2,37E+11**	**2,6E+11**
**SD**	3,91E+10	2,86E+10	2,4E+10	3,1E+10
**CI (95%)**	**2,42E+10**	**1,77E+10**	**1,49E+10**	**1,9E+10**
**LL**	3,03E+11	2,07E+11	2,22E+11	2,4E+11
**UL**	3,52E+11	2,43E+11	2,52E+11	2,8E+11
**Size (Mean)**	115 nm	91,7 nm	127,1 nm	122 nm
**Size (Mode)**	89,6 nm	70,9 nm	95 nm	91 nm

Supplementary Table 2: Proteins identified exclusively in convalescent plasma derived exosomes

Uniprot Accession	Protein names
Q8NF17	FLJ00385 protein (Fragment)
P07360	Complement component C8 gamma chain
A2NUT2	Lambda-chain (AA -20 to 215)
P00736	Complement C1r subcomponent (EC 3.4.21.41) (Complement component 1 subcomponent r) [Cleaved into: Complement C1r subcomponent heavy chain; Complement C1r subcomponent light chain]
D1MGQ2	Alpha-1-globin (Alpha-2 globin chain) (Delta globin) (HCG1745306, isoform CRA_b) (Mutant hemoglobin subunit alpha 1)
P19823	Inter-alpha-trypsin inhibitor heavy chain H2 (ITI heavy chain H2) (ITI-HC2) (Inter-alpha-inhibitor heavy chain 2) (Inter-alpha-trypsin inhibitor complex component II) (Serum-derived hyaluronan-associated protein) (SHAP)
B4DGC3	Apolipoprotein D
I2D5I8	Apolipoprotein M (Fragment)

B4E1D8	cDNA FLJ51597, highly similar to C4b-binding protein alpha chain
B7ZA94	Complement component 8 subunit beta (Complement component C8 beta chain)
D9IWP9	Apolipoprotein H (Beta-2-glycoprotein 1) (Beta-2-glycoprotein I) (Fragment)
B4DUV1	Fibulin-1
U5LKN4	Apolipoprotein L1 (Fragment)
A2KBC3	Anti-(ED-B) scFV (Fragment)
B7Z6N2	Actin-depolymerizing factor (Brevin) (Gelsolin)
A0A087WT59	Transthyretin
D3DNU8	Kininogen 1, isoform CRA_a
E9M4D4	Hemoglobin alpha-1 globin chain (Fragment)
A2KBC7	Anti-IFN-G scFv (Fragment)
B2R8I2	cDNA, FLJ93914, highly similar to Homo sapiens histidine-rich glycoprotein (HRG), mRNA
P00450	Ceruloplasmin (EC 1.16.3.1) (Ferroxidase)
Q05CV2	C8A protein (Fragment)
P02751	Fibronectin (FN) (Cold-insoluble globulin) (CIG) [Cleaved into: Anastellin; Ugl-Y1; Ugl-Y2; Ugl-Y3]
A6XGL1	Transthyretin
B1AHL2	Fibulin-1
G3V595	Alpha-1-antichymotrypsin (Fragment)
B3KNX0	cDNA FLJ30621 fis, clone CTONG2001681, highly similar to Complement C1s subcomponent
B2R6W1	cDNA, FLJ93143, highly similar to Homo sapiens complement component 7 (C7), mRNA
P07358	Complement component C8 beta chain (Complement component 8 subunit beta)
U5LKJ0	Apolipoprotein L1 (Fragment)
B4E1B0	Complement subcomponent C1r (EC 3.4.21.41)
Q53HT9	Complement subcomponent C1r (EC 3.4.21.41) (Fragment)
Q02224	Centromere-associated protein E (Centromere protein E) (CENP-E) (Kinesin-7) (Kinesin-related protein CENPE)
Q6N093	Uncharacterized protein DKFZp686I04196 (Fragment)
B4DZM1	cDNA FLJ58310, highly similar to Homo sapiens trinucleotide repeat containing 15 (TNRC15), mRNA
H7C5N4	Coiled-coil domain-containing protein 52 (Spindle and centriole-associated protein 1) (Fragment)
B7Z550	Complement component 8 subunit beta (Complement component C8 beta chain)
P01023	Alpha-2-macroglobulin (Alpha-2-M) (C3 and PZP-like alpha-2-macroglobulin domain-containing protein 5)
U5LKG0	Apolipoprotein L1 (Fragment)
U5LGU2	Apolipoprotein L1 (Fragment)
B4DPN0	Apolipoprotein H (Beta-2-glycoprotein 1) (Beta-2-glycoprotein I)
U5LIC5	Apolipoprotein L1 (Fragment)
E9PDH4	1-phosphatidylinositol 3-phosphate 5-kinase (Fragment)
B7Z2X4	Actin-depolymerizing factor (Brevin) (Gelsolin)
P07357	Complement component C8 alpha chain (Complement component 8 subunit alpha)
P07225	Vitamin K-dependent protein S
I1VZV6	Hemoglobin alpha 1
Q8N355	IGL@ protein

E1A689	Mutant Apo B 100
A0A087WV45	Transthyretin (Fragment)
A2KBC6	Anti-FactorVIII scFv (Fragment)
Q7Z5E2	Medulloblastoma antigen MU-MB-50.72 (Fragment)
F8WAS2	Inter-alpha-trypsin inhibitor heavy chain H1
E9PAQ1	Properdin
B4E1N5	cDNA FLJ53694, highly similar to Apolipoprotein-L1
C9J7V5	Properdin (Fragment)
B7Z5Q2	cDNA FLJ58075, highly similar to Ceruloplasmin
A0N5G5	Rheumatoid factor D5 light chain (Fragment)
A2KBC2	Anti-(ED-B) scFV (Fragment)
A2NB45	Cold agglutinin FS-1 L-chain (Fragment)
U5LKH9	Apolipoprotein L1 (Fragment)
F8WCZ6	Complement C1s subcomponent
S6AWE6	IgG L chain
P08603	Complement factor H (H factor 1)
A5PL32	APOL1 protein (Fragment)
C9JX71	Apolipoprotein D (Fragment)
U5LI90	Apolipoprotein L1 (Fragment)
B7Z9A0	Actin-depolymerizing factor (Brevin) (Gelsolin)
Q5JXL1	PHD finger protein 20 (Fragment)
A8K2N0	cDNA FLJ77835, highly similar to Homo sapiens complement component 1, s subcomponent (C1S), transcript variant 2, mRNA
A0A087X232	Complement C1s subcomponent
Q5EBM2	Uncharacterized protein
D9ZGG2	Vitronectin
P00751	Complement factor B (EC 3.4.21.47) (C3/C5 convertase) (Glycine-rich beta glycoprotein) (GBG) (PBF2) (Properdin factor B) [Cleaved into: Complement factor B Ba fragment; Complement factor B Bb fragment]
B4DM79	cDNA FLJ53848, highly similar to Inter-alpha-trypsin inhibitor heavy chain H2
U3PXP0	Alpha globin chain (Fragment)
P0C0L4	Complement C4-A (Acidic complement C4) (C3 and PZP-like alpha-2-macroglobulin domain-containing protein 2) [Cleaved into: Complement C4 beta chain; Complement C4-A alpha chain; C4a anaphylatoxin; C4b-A; C4d-A; Complement C4 gamma chain]
B4DNT5	cDNA FLJ60316, highly similar to Apolipoprotein-L1
A0A087X0P0	Centromere-associated protein E
F5H7G1	Complement component 8 subunit beta (Complement component C8 beta chain)
V9GYG9	Apolipoprotein A-II (Apolipoprotein A2) (Fragment)
Q8N0Z3	Spindle and centriole-associated protein 1 (Coiled-coil domain-containing protein 52) (Spindle and centriole-associated protein)
Q5JWZ0	PHD finger protein 20 (Fragment)
A0A0B4J1Y9	Immunoglobulin heavy variable 3-72
Q6PIL8	IGK@ protein
A0A024R1Q4	Apolipoprotein L, 1, isoform CRA_c
B2RMS9	Inter-alpha (Globulin) inhibitor H4 (Plasma Kallikrein-sensitive glycoprotein)
D6RD17	Immunoglobulin J chain (Fragment)

E9PIT3	Activation peptide fragment 1 (EC 3.4.21.5) (Activation peptide fragment 2) (Coagulation factor II) (Prothrombin) (Thrombin heavy chain) (Thrombin light chain)
B3KS79	cDNA FLJ35730 fis, clone TESTI2003131, highly similar to ALPHA-1-ANTICHYMOTRYPSIN
P04003	C4b-binding protein alpha chain (C4bp) (Proline-rich protein) (PRP)
P02748	Complement component C9 [Cleaved into: Complement component C9a; Complement component C9b]
O14791	Apolipoprotein L1 (Apolipoprotein L) (Apo-L) (ApoL) (Apolipoprotein L-I) (ApoL-I)
P0C0L5	Complement C4-B (Basic complement C4) (C3 and PZP-like alpha-2-macroglobulin domain-containing protein 3) [Cleaved into: Complement C4 beta chain; Complement C4-B alpha chain; C4a anaphylatoxin; C4b-B; C4d-B; Complement C4 gamma chain]
P06727	Apolipoprotein A-IV (Apo-AIV) (ApoA-IV) (Apolipoprotein A4)
Q8NBH6	Fibulin-1
B3KVK6	Complement factor properdin, isoform CRA_c (cDNA FLJ16673 fis, clone THYMU3003403, highly similar to Properdin)
B4DPQ0	Complement subcomponent C1r (EC 3.4.21.41)
V9HWA9	C3-beta-c (C3a anaphylatoxin) (Complement C3) (Complement C3 alpha chain) (Complement C3 beta chain) (Complement C3b alpha' chain) (Complement C3c alpha' chain fragment 1) (Complement C3c alpha' chain fragment 2) (Complement C3d fragment) (Complement C3dg fragment) (Complement C3f fragment) (Complement C3g fragment)
Q2KHQ6	APOL1 protein (Apolipoprotein L, 1)
A2KBC5	Anti-HCS scFv (Fragment)
Q3T8J9	GON-4-like protein (GON-4 homolog)
A6XMH1	Transthyretin
V9HWD8	Epididymis secretory sperm binding protein Li 163pA
A0A024R944	Antithrombin-III (Serpin C1)
Q1WWL5	APOL1 protein (Fragment)
A0A024R462	Fibronectin
B7Z539	cDNA FLJ56954, highly similar to Inter-alpha-trypsin inhibitor heavy chain H1
B2R9E5	cDNA, FLJ94353, highly similar to Homo sapiens apolipoprotein L, 1 (APOL1), transcript variant 2, mRNA
Q16519	Vitamin K-dependent protein S (Fragment)
P05090	Apolipoprotein D (Apo-D) (ApoD)
U5LGY0	Apolipoprotein L1 (Fragment)
O95445	Apolipoprotein M (Apo-M) (ApoM) (Protein G3a)
Q5T0H8	Gelsolin (Fragment)
Q0ZCH9	Immunglobulin heavy chain variable region (Fragment)
U5LIG7	Apolipoprotein L1 (Fragment)
D6RF35	Gc-globulin (Group-specific component) (Vitamin D-binding protein)
A2KBC0	Anti-(ED-B) scFV (Fragment)
F5H2D0	Complement subcomponent C1r (EC 3.4.21.41)
A0A024R1G8	Apolipoprotein L, 1, isoform CRA_b
V9HWI6	Gc-globulin (Group-specific component) (Vitamin D-binding protein)
P01860	Immunoglobulin heavy constant gamma 3 (HDC) (Heavy chain disease protein) (Ig gamma-3 chain C region)
Q14686	Nuclear receptor coactivator 6 (Activating signal cointegrator 2) (ASC-2) (Amplified in breast cancer protein 3) (Cancer-amplified transcriptional coactivator ASC-2) (Nuclear

	receptor coactivator RAP250) (NRC RAP250) (Nuclear receptor-activating protein, 250 kDa) (Peroxisome proliferator-activated receptor-interacting protein) (PPAR-interacting protein) (PRIP) (Thyroid hormone receptor-binding protein)
D3DRR6	Inter-alpha (Globulin) inhibitor H2, isoform CRA_a
Q1L857	Ceruloplasmin (Fragment)
D6R934	Complement C1q subcomponent subunit B
L8E853	von Willebrand factor
U5LKP5	Apolipoprotein L1 (Fragment)
Q9UL88	Myosin-reactive immunoglobulin heavy chain variable region (Fragment)
Q6N030	Uncharacterized protein
A6NMB1	Sialic acid-binding Ig-like lectin 16 (Siglec-16) (Siglec-P16)
P09871	Complement C1s subcomponent (EC 3.4.21.42) (C1 esterase) (Complement component 1 subcomponent s) [Cleaved into: Complement C1s subcomponent heavy chain; Complement C1s subcomponent light chain]
P20851	C4b-binding protein beta chain
U5LH96	Apolipoprotein L1 (Fragment)
P23142	Fibulin-1 (FIBL-1)
F5GY80	Complement component 8 subunit beta (Complement component C8 beta chain)
U5LHF6	Apolipoprotein L1 (Fragment)
A8K5T0	cDNA FLJ75416, highly similar to Homo sapiens complement factor H (CFH), mRNA
B4E1Z4	C3/C5 convertase (EC 3.4.21.47) (Complement factor B) (Complement factor B Ba fragment) (Complement factor B Bb fragment)
U5LII8	Apolipoprotein L1 (Fragment)
S6B2B0	IgG L chain
P01717	Immunoglobulin lambda variable 3-25 (Ig lambda chain V-IV region Hil)
Q5SRP5	Apolipoprotein M
U6A216	Mutant hemoglobin alpha 1 globin chain (Mutant hemoglobin alpha 2 globin chain) (Fragment)
Q9Y2I7	1-phosphatidylinositol 3-phosphate 5-kinase (Phosphatidylinositol 3-phosphate 5-kinase) (EC 2.7.1.150) (FYVE finger-containing phosphoinositide kinase) (PIKfyve) (Phosphatidylinositol 3-phosphate 5-kinase type III) (PIPkin-III) (Type III PIP kinase) (Serine-protein kinase PIKFYVE) (EC 2.7.11.1)
Q05CF8	KNG1 protein
B7Z549	cDNA FLJ56821, highly similar to Inter-alpha-trypsin inhibitor heavy chain H1
U5LIJ1	Apolipoprotein L1 (Fragment)
Q2KHM9	Protein moonraker (MNR) (OFD1- and FOPNL-interacting protein)
Q9UL78	Myosin-reactive immunoglobulin light chain variable region (Fragment)
Q6ZNP0	cDNA FLJ27417 fis, clone WMC06579
P19827	Inter-alpha-trypsin inhibitor heavy chain H1 (ITI heavy chain H1) (ITI-HC1) (Inter-alpha-inhibitor heavy chain 1) (Inter-alpha-trypsin inhibitor complex component III) (Serum-derived hyaluronan-associated protein) (SHAP)
P02747	Complement C1q subcomponent subunit C
P01619	Immunoglobulin kappa variable 3-20 (Ig kappa chain V-III region B6) (Ig kappa chain V-III region GOL) (Ig kappa chain V-III region HAH) (Ig kappa chain V-III region HIC) (Ig kappa chain V-III region IARC/BL41) (Ig kappa chain V-III region NG9) (Ig kappa chain V-III region SIE) (Ig kappa chain V-III region Ti) (Ig kappa chain V-III region WOL)
S6B286	IgG L chain

Q6ZS32	cDNA FLJ45870 fis, clone OCBBF3005330, highly similar to FYVE finger-containing phosphoinositide kinase (Fragment)
E9PFZ2	Ceruloplasmin
V9H1D9	Alpha globin
U5LGW0	Apolipoprotein L1 (Fragment)
A0A0A0MT01	Actin-depolymerizing factor (Brevin) (Gelsolin)
A2MYD4	V2-7 protein (Fragment)
A0A5E4	Uncharacterized protein
U5LH46	Apolipoprotein L1 (Fragment)
Q59GS8	Complement component 5 variant (Fragment)
Q5NV90	V2-17 protein (Fragment)
B4DPP8	cDNA FLJ53075, highly similar to Kininogen-1
P02790	Hemopexin (Beta-1B-glycoprotein)
Q5T985	Inter-alpha-trypsin inhibitor heavy chain H2
V9HWG4	1-phosphatidylinositol-3-phosphate 5-kinase (EC 2.7.1.150)
I2D5J2	Apolipoprotein M (Fragment)
P27918	Properdin (Complement factor P)
A0A024R962	HCG40889, isoform CRA_b
B7Z992	Actin-depolymerizing factor (Brevin) (Gelsolin)
B7Z555	Complement component 8 subunit beta (Complement component C8 beta chain)
Q53HU9	Complement subcomponent C1r (EC 3.4.21.41) (Fragment)
Q96PD5	N-acetylmuramoyl-L-alanine amidase (EC 3.5.1.28) (Peptidoglycan recognition protein 2) (Peptidoglycan recognition protein long) (PGRP-L)
P10643	Complement component C7
P01591	Immunoglobulin J chain (Joining chain of multimeric IgA and IgM)
C9JF17	Apolipoprotein D (Fragment)
Q0ZCI9	Immunglobulin heavy chain variable region (Fragment)
B7Z544	cDNA FLJ51742, highly similar to Inter-alpha-trypsin inhibitor heavy chain H4
O43866	CD5 antigen-like (Apoptosis inhibitor expressed by macrophages) (hAIM) (CT-2) (IgM-associated peptide) (SP-alpha)
A8K5J8	Complement subcomponent C1r (EC 3.4.21.41)
U5LKM6	Apolipoprotein L1 (Fragment)
C0JYY2	Apolipoprotein B (Including Ag(X) antigen) (Apolipoprotein B (Including Ag(X) antigen), isoform CRA_a)
A0A024R6I7	Alpha-1-antitrypsin (Serpin peptidase inhibitor, clade A (Alpha-1 antiproteinase, antitrypsin), member 1, isoform CRA_a)
Q6GMX4	IGL@ protein
P06396	Gelsolin (AGEL) (Actin-depolymerizing factor) (ADF) (Brevin)
P25311	Zinc-alpha-2-glycoprotein (Zn-alpha-2-GP) (Zn-alpha-2-glycoprotein)
C9JPV4	Alpha-2-antiplasmin (Fragment)
Q9NSD0	Vitamin K-dependent protein S
P10909	Clusterin (Aging-associated gene 4 protein) (Apolipoprotein J) (Apo-J) (Complement cytolysis inhibitor) (CLI) (Complement-associated protein SP-40,40) (Ku70-binding protein 1) (NA1/NA2) (Sulfated glycoprotein 2) (SGP-2) (Testosterone-repressed prostate message 2) (TRPM-2) [Cleaved into: Clusterin beta chain (ApoJalpha) (Complement cytolysis inhibitor a chain); Clusterin alpha chain (ApoJbeta) (Complement cytolysis inhibitor b chain)]

U5LKT7	Apolipoprotein L1 (Fragment)
L8E7T8	Alternative protein ZNF256
P08697	Alpha-2-antiplasmin (Alpha-2-AP) (Alpha-2-plasmin inhibitor) (Alpha-2-PI) (Serpin F2)
P13671	Complement component C6
A8K008	Uncharacterized protein
P00747	Plasminogen (EC 3.4.21.7) [Cleaved into: Plasmin heavy chain A; Activation peptide; Angiostatin; Plasmin heavy chain A, short form; Plasmin light chain B]
A2KBC4	Anti-TN-C scFv (Fragment)
B7ZKJ8	ITIH4 protein (Inter-alpha-trypsin inhibitor heavy chain H4)
A8K9M5	Complement component 8 subunit beta (Complement component C8 beta chain)
P22792	Carboxypeptidase N subunit 2 (Carboxypeptidase N 83 kDa chain) (Carboxypeptidase N large subunit) (Carboxypeptidase N polypeptide 2) (Carboxypeptidase N regulatory subunit)
A8K8Z4	Complement component C6
U5LKN0	Apolipoprotein L1 (Fragment)
Q9BVI0	PHD finger protein 20 (Glioma-expressed antigen 2) (Hepatocellular carcinoma-associated antigen 58) (Novel zinc finger protein) (Transcription factor TZP)
Q14624	Inter-alpha-trypsin inhibitor heavy chain H4 (ITI heavy chain H4) (ITI-HC4) (Inter-alpha-inhibitor heavy chain 4) (Inter-alpha-trypsin inhibitor family heavy chain-related protein) (IHRP) (Plasma kallikrein sensitive glycoprotein 120) (Gp120) (PK-120) [Cleaved into: 70 kDa inter-alpha-trypsin inhibitor heavy chain H4; 35 kDa inter-alpha-trypsin inhibitor heavy chain H4]
P06312	Immunoglobulin kappa variable 4-1 (Ig kappa chain V-IV region B17) (Ig kappa chain V-IV region JI) (Ig kappa chain V-IV region Len) (Ig kappa chain V-IV region STH)
A0A0A0MS51	Actin-depolymerizing factor (Brevin) (Gelsolin)
B2R7F8	Plasminogen (EC 3.4.21.7)
P01009	Alpha-1-antitrypsin (Alpha-1 protease inhibitor) (Alpha-1-antiproteinase) (Serpin A1) [Cleaved into: Short peptide from AAT (SPAAT)]
A0A0A0MSV6	Complement C1q subcomponent subunit B (Fragment)
A0A024R035	Complement component C9
P02746	Complement C1q subcomponent subunit B
S6BGF9	IgG L chain
A6NJZ3	Olfactory receptor 6C65
A2KBC8	Anti-TeTox scFv (Fragment)
Q6MZV7	Uncharacterized protein DKFZp686C11235
Q9BX83	Hemoglobin alpha 1 globin chain (Fragment)
C9JMH6	Alpha-2-antiplasmin (Fragment)
P02763	Alpha-1-acid glycoprotein 1 (AGP 1) (Orosomucoid-1) (OMD 1)
P01615	Immunoglobulin kappa variable 2D-28 (Ig kappa chain V-II region FR) (Ig kappa chain V-II region GM607) (Ig kappa chain V-II region MIL) (Ig kappa chain V-II region TEW)
U5LIH6	Apolipoprotein L1 (Fragment)
P01861	Immunoglobulin heavy constant gamma 4 (Ig gamma-4 chain C region)
Q29RW7	APOL1 protein (Fragment)
P01767	Immunoglobulin heavy variable 3-53 (Ig heavy chain V-III region BUT)
Q4ZGM8	Hemoglobin alpha-2 globin mutant (Fragment)
B0QYY0	PHD finger protein 20 (Fragment)
Q6GMV8	Uncharacterized protein
U5LKR8	Apolipoprotein L1 (Fragment)

Q6P5S8	IGK@ protein
P01031	Complement C5 (C3 and PZP-like alpha-2-macroglobulin domain-containing protein 4) [Cleaved into: Complement C5 beta chain; Complement C5 alpha chain; C5a anaphylatoxin; Complement C5 alpha' chain]
F5GXS0	C4a anaphylatoxin (Complement C4 gamma chain)
E1B2D1	Hemoglobin alpha-1 globin chain variant (Fragment)
P02749	Beta-2-glycoprotein 1 (APC inhibitor) (Activated protein C-binding protein) (Anticardiolipin cofactor) (Apolipoprotein H) (Apo-H) (Beta-2-glycoprotein I) (B2GPI) (Beta(2)GPI)
Q05CV3	Complement component 8 subunit beta (Complement component C8 beta chain) (Fragment)
A8K5A4	cDNA FLJ76826, highly similar to Homo sapiens ceruloplasmin (ferroxidase) (CP), mRNA
E9KL36	Transthyretin
B4E1E1	cDNA FLJ51598, highly similar to C4b-binding protein alpha chain
Q6NS95	IGL@ protein
A2RTY6	Inter-alpha (Globulin) inhibitor H2 (Inter-alpha (Globulin) inhibitor H2, isoform CRA_b) (cDNA FLJ75038, highly similar to Homo sapiens inter-alpha (globulin) inhibitor H2 (ITIH2), mRNA)
P02760	Protein AMBP [Cleaved into: Alpha-1-microglobulin (Protein HC) (Alpha-1 microglycoprotein) (Complex-forming glycoprotein heterogeneous in charge); Inter-alpha-trypsin inhibitor light chain (ITI-LC) (Bikunin) (EDC1) (HI-30) (Uronic-acid-rich protein); Trypstatin]
A2KBB9	Anti-(ED-B) scFV (Fragment)
Q5NV69	V1-13 protein (Fragment)
P04114	Apolipoprotein B-100 (Apo B-100) [Cleaved into: Apolipoprotein B-48 (Apo B-48)]
U5LKC6	Apolipoprotein L1 (Fragment)
J3QLI0	Beta-2-glycoprotein 1 (Fragment)
P00734	Prothrombin (EC 3.4.21.5) (Coagulation factor II) [Cleaved into: Activation peptide fragment 1; Activation peptide fragment 2; Thrombin light chain; Thrombin heavy chain]
B7Z4U6	Actin-depolymerizing factor (Brevin) (Gelsolin)
P04275	von Willebrand factor (vWF) [Cleaved into: von Willebrand antigen 2 (von Willebrand antigen II)]
A0A024R6P0	Serpin peptidase inhibitor, clade A (Alpha-1 antiproteinase, antitrypsin), member 3, isoform CRA_c
P01834	Immunoglobulin kappa constant (Ig kappa chain C region) (Ig kappa chain C region AG) (Ig kappa chain C region CUM) (Ig kappa chain C region EU) (Ig kappa chain C region OU) (Ig kappa chain C region ROY) (Ig kappa chain C region TI)
U5LIA9	Apolipoprotein L1 (Fragment)
B3KS49	Actin-depolymerizing factor (Brevin) (Gelsolin)
P04196	Histidine-rich glycoprotein (Histidine-proline-rich glycoprotein) (HPRG)
U5LKQ0	Apolipoprotein L1 (Fragment)

Supplementary Table 3: Proteins found in both healthy and convalescent plasma derived exosomes

,

Uniprot Accession	Protein names
S6BGD6	IgG L chain
Q6PIQ7	IGL@ protein
V9HVY1	Fibrinogen beta chain (Fibrinopeptide B)
P02647	Apolipoprotein A-I (Apo-AI) (ApoA-I) (Apolipoprotein A1) [Cleaved into: Proapolipoprotein A-I (ProapoA-I); Truncated apolipoprotein A-I (Apolipoprotein A-I(1-242))]
C9JC84	Fibrinogen gamma chain
V9GYM3	Apolipoprotein A-II (Apolipoprotein A2)
P02649	Apolipoprotein E (Apo-E)
P01876	Immunoglobulin heavy constant alpha 1 (Ig alpha-1 chain C region) (Ig alpha-1 chain C region BUR) (Ig alpha-1 chain C region TRO)
P02671	Fibrinogen alpha chain [Cleaved into: Fibrinopeptide A; Fibrinogen alpha chain]
P01714	Immunoglobulin lambda variable 3-19 (Ig lambda chain V-III region SH)
B7Z8Q2	Alpha-2-HS-glycoprotein (Fetuin-A)
J9ZVQ3	Apolipoprotein E (Fragment)
Q6MZW0	Uncharacterized protein DKFZp686J11235 (Fragment)
P02652	Apolipoprotein A-II (Apo-AII) (ApoA-II) (Apolipoprotein A2) [Cleaved into: Proapolipoprotein A-II (ProapoA-II); Truncated apolipoprotein A-II (Apolipoprotein A-II(1-76))]
P02765	Alpha-2-HS-glycoprotein (Alpha-2-Z-globulin) (Ba-alpha-2-glycoprotein) (Fetuin-A) [Cleaved into: Alpha-2-HS-glycoprotein chain A; Alpha-2-HS-glycoprotein chain B]
V9GYE3	Apolipoprotein A-II (Apolipoprotein A2)
Q96K68	cDNA FLJ14473 fis, clone MAMMA1001080, highly similar to Homo sapiens SNC73 protein (SNC73) mRNA
Q53H26	Beta-1 metal-binding globulin (Serotransferrin) (Siderophilin) (Fragment)
B4E1B2	Beta-1 metal-binding globulin (Serotransferrin) (Siderophilin)
Q8NEJ1	Uncharacterized protein
Q6ZVX0	cDNA FLJ41981 fis, clone SMINT2011888, highly similar to Protein Tro alpha1 H,myeloma
Q9UP60	SNC73 protein
P00738	Haptoglobin (Zonulin) [Cleaved into: Haptoglobin alpha chain; Haptoglobin beta chain]
Q8NCL6	cDNA FLJ90170 fis, clone MAMMA1000370, highly similar to Ig alpha-1 chain C region
C9JEU5	Fibrinogen gamma chain
P02787	Serotransferrin (Transferrin) (Beta-1 metal-binding globulin) (Siderophilin)
P02768	Albumin
P02679	Fibrinogen gamma chain
C9JV77	Alpha-2-HS-glycoprotein (Fetuin-A)
Q5FWF9	IGL@ protein

Supplementary Table 4: Proteins found in ony healthy plasma derived exosomes

Uniprot Accession	Protein names

P02656	Apolipoprotein C-III (Apo-CIII) (ApoC-III) (Apolipoprotein C3)
A0A024R0T8	Apolipoprotein C-I, isoform CRA_a
Q7Z379	Uncharacterized protein DKFZp686K04218 (Fragment)
B7WNR0	Albumin
K7EPF9	Apolipoprotein C-I
A8K9P0	cDNA FLJ78413, highly similar to Homo sapiens albumin, mRNA
Q06AH7	Beta-1 metal-binding globulin (Serotransferrin) (Siderophilin)
H0YA55	Albumin (Fragment)
B4DHZ6	Transferrin, isoform CRA_c (cDNA FLJ54029, highly similar to Serotransferrin)
B2RBS8	cDNA, FLJ95666, highly similar to Homo sapiens albumin (ALB), mRNA
F5H7V9	Tenascin
Q4LE33	TNC variant protein (Fragment)
H0Y300	Haptoglobin (Haptoglobin alpha chain) (Haptoglobin beta chain)
B4DPP6	cDNA FLJ54371, highly similar to Serum albumin
Q9NPP6	Immunoglobulin heavy chain variant (Fragment)
Q96DK0	cDNA FLJ25298 fis, clone STM07683, highly similar to Protein Tro alpha1 H,myeloma
Q6N090	Uncharacterized protein DKFZp686G21220 (Fragment)
K7ELM9	Apolipoprotein C-I
P24821	Tenascin (TN) (Cytotactin) (GMEM) (GP 150-225) (Glioma-associated-extracellular matrix antigen) (Hexabrachion) (JI) (Myotendinous antigen) (Neuronectin) (Tenascin-C) (TN-C)
Q7Z2U7	Uncharacterized protein
P01877	Immunoglobulin heavy constant alpha 2 (Ig alpha-2 chain C region) (Ig alpha-2 chain C region BUT) (Ig alpha-2 chain C region LAN)
Q6MZV6	Uncharacterized protein DKFZp686L19235
B0YIW2	Apolipoprotein C-III (Apolipoprotein C3)
Q6MZX9	Uncharacterized protein DKFZp686M08189
Q7Z374	Uncharacterized protein DKFZp686C02218 (Fragment)
A0A087WWT3	Albumin
J3QSU6	Tenascin
Q8IWB1	Inositol 1,4,5-trisphosphate receptor-interacting protein (Protein DANGER)
P0DOY2	Immunoglobulin lambda constant 2 (Ig lambda chain C region Kern) (Ig lambda chain C region NIG-64) (Ig lambda chain C region SH) (Ig lambda chain C region X) (Ig lambda-2 chain C region)
Q8WY24	SNC66 protein
Q6GMW3	IGL@ protein
Q6N092	Uncharacterized protein DKFZp686K18196 (Fragment)
D3YHM4	Tenascin-C isoform 14/AD1/16
A0A024R884	Tenascin C (Hexabrachion), isoform CRA_a
K7ERI9	Apolipoprotein C-I (Fragment)
P0DOY3	Immunoglobulin lambda constant 3 (Ig lambda chain C region DOT) (Ig lambda chain C region NEWM) (Ig lambda-3 chain C regions)
Q6ZW64	cDNA FLJ41552 fis, clone COLON2004478, highly similar to Protein Tro alpha1 H,myeloma
F6KPG5	Albumin (Fragment)
C9JKR2	Albumin (Albumin, isoform CRA_k)

Q6P089	IGH@ protein
D6RHD5	Albumin
J3QR68	Haptoglobin (Haptoglobin alpha chain) (Haptoglobin beta chain) (Fragment)
Q56G89	Serum albumin
B4DI57	cDNA FLJ54111, highly similar to Serotransferrin
B4DPR2	cDNA FLJ50830, highly similar to Serum albumin
K7EJI9	Apolipoprotein C-I
Q567P1	IGL@ protein
H0YGZ3	Tenascin (Fragment)
Q8TCJ5	Uncharacterized protein DKFZp667J0810 (Fragment)

Supplementary Table 5: Proteins associated with the KO Term "Coronavirus disease - COVID-19"

Accession Numbers	Protein Names
P00734	THRB_HUMAN
P01031	CO5_HUMAN
P02746	C1QB_HUMAN
P04275	VWF_HUMAN
P07357	CO8A_HUMAN
P07358	CO8B_HUMAN
P0C0L5	CO4B_HUMAN
P10643	CO7_HUMAN
A0A024R035	A0A024R035_HUMAN
A0A087X232	A0A087X232_HUMAN
A0A0A0MSV6	A0A0A0MSV6_HUMAN
A2KBC6	A2KBC6_HUMAN
A2KBC7	A2KBC7_HUMAN
A8K2N0	A8K2N0_HUMAN
A8K5J8	A8K5J8_HUMAN
A8K8Z4	A8K8Z4_HUMAN
B4DPQ0	B4DPQ0_HUMAN
B4E1B0	B4E1B0_HUMAN
B4E1Z4	B4E1Z4_HUMAN
B7Z550	B7Z550_HUMAN
F5GXS0	F5GXS0_HUMAN
F5H2D0	F5H2D0_HUMAN
F5H7G1	F5H7G1_HUMAN
F8WCZ6	F8WCZ6_HUMAN
Q53HT9	Q53HT9_HUMAN
Q53HU9	Q53HU9_HUMAN
Q6N093	Q6N093_HUMAN
P00736	C1R_HUMAN
P00751	CFAB_HUMAN
P02747	C1QC_HUMAN
P02748	CO9_HUMAN

P07360	CO8G_HUMAN
P0C0L4	CO4A_HUMAN
P13671	CO6_HUMAN
A2KBC3	A2KBC3_HUMAN
B2R6W1	B2R6W1_HUMAN
B3KNX0	B3KNX0_HUMAN
B7ZA94	B7ZA94_HUMAN
D6R934	D6R934_HUMAN
E9PIT3	E9PIT3_HUMAN
L8E853	L8E853_HUMAN
Q05CV2	Q05CV2_HUMAN
Q59GS8	Q59GS8_HUMAN
V9HWA9	V9HWA9_HUMAN
Q9UL88	Q9UL88_HUMAN
Q6N030	Q6N030_HUMAN
Q0ZCI9	Q0ZCI9_HUMAN
A8K008	A8K008_HUMAN
A2KBC4	A2KBC4_HUMAN
A2KBC8	A2KBC8_HUMAN
Q6MZV7	Q6MZV7_HUMAN
P01767	HV353_HUMAN
A2KBB9	A2KBB9_HUMAN
A2KBC0	A2KBC0_HUMAN
Q0ZCH9	Q0ZCH9_HUMAN
A2KBC5	A2KBC5_HUMAN
A2KBC2	A2KBC2_HUMAN
Q5EBM2	Q5EBM2_HUMAN
P09871	C1S_HUMAN
F5GY80	F5GY80_HUMAN
B7Z555	B7Z555_HUMAN
A0A0B4J1Y9	HV372_HUMAN
A8K9M5	A8K9M5_HUMAN
Q05CV3	Q05CV3_HUMAN
